# Anti-Inflammatory Oleanolic Triterpenes from Chinese Acorns

**DOI:** 10.3390/molecules21050669

**Published:** 2016-05-20

**Authors:** Jie Huang, Yihai Wang, Chuan Li, Xinluan Wang, Xiangjiu He

**Affiliations:** 1 School of Pharmacy, Guangdong Pharmaceutical University, Guangzhou 510006, China; hj_yellow@163.com (J.H.); wangyihai@whu.edu.cn (Y.W.); inuyashachuanli@163.com (C.L.); 2 Shenzhen Institute of Advanced Technology, Chinese Academy of Sciences, Shenzhen 518055, China; xl.wang@siat.ac.cn

**Keywords:** Chinese acorns, oleanolic triterpenes, *Quercus serrata* var. brevipetiolata, anti-inflammatory activity, NO production, IL-6, IL-8

## Abstract

Acorns play an important role in human history and are a source of food and recipes for many cultures around the world. In this study, eleven oleanolic triterpenes, one of which was novel, were isolated from Chinese acorns (*Quercus serrata* var. brevipetiolata). The chemical structure of the novel triterpene, which was identified as 2α,3β,19α-trihydroxy-24-oxo-olean-12-en-28-oic acid (**1**), was established based on the interpretation of chemical and spectroscopic analyses, including IR, HR-ESI-MS, and NMR experiments (^1^H, ^13^C NMR, DEPT, ^1^H-^1^H COSY, HSQC, HMBC, and NOESY). All isolated compounds were tested for their inhibitory effects on LPS-induced nitric oxide (NO) production in RAW 264.7 macrophages. Compared with the positive control drug indomethacin (IC_50_ = 47.4 μM), compounds **1**, **3**, **6** and **8** exhibited remarkable anti-inflammatory activities with IC_50_ values of 5.4, 7.8, 4.0 and 8.9 μM, respectively. Besides, compounds **2**, **4**, **7** and **9** also showed moderate anti-inflammatory activities with IC_50_ values of 10.1, 13.0, 20.1 and 17.2 μM, respectively. Furthermore, Compound **1** could inhibit TNF-α-induced IL-6 and IL-8 production in MH7A cells.

## 1. Introduction

The oaks genus, with the scientific name *Quercus*, is broadly distributed in the Northern Hemisphere, and has been announced to become the national tree of the United States of America [1]. Besides North America, the second greatest center of oak diversity is China, which has approximately 100 species [2]. The acorn, which is the nut of the oak, usually contains a single seed (occasionally two), enclosed in a tough, leathery shell, and borne in a cup-shaped cupule. It’s an important natural resource because of its ubiquity and the role of many of its species in food production, livestock and poultry breeding [3]. As reported, acorns are rich in important nutrients, such as carbohydrates, proteins and fatty acids, as well as antioxidant compounds, namely phenolics and sterols [4,5,6]. In traditional Chinese medicine, acorns are mainly used to treat diarrhea, evil sores, scrofula, *etc.* [7]. The acorn extracts have been used in folk medicine as treatment for several inflammatory diseases [8].

Inflammation is a host defense response to injury or bacterial/viral infections, which is accompanied by production of a series of inflammatory mediators, such as NO, TNF- α, IL-1β, and IL-6 [9]. It is a protective response that involves immune cells, blood vessels, and molecular mediators. The purpose of inflammation is to eliminate the initial cause of cell injury, clear out necrotic cells and tissues damaged from the original insult and the inflammatory process, and to initiate tissue repair. However, acute and chronic inflammation may lead to a host of diseases, such as hay fever, periodontitis, atherosclerosis, rheumatoid arthritis, and even cancer. Nonsteroidal anti-inflammatory drugs (NASIDs) are a common kind of anti-inflammatory drug. However, as ongoing research shows, more and more adverse effects have been found during their clinical application. Use of NSAIDs increases the risk of having a range of gastrointestinal problems. When NSAIDs are used for pain management after surgery they cause increased risk of kidney and liver problems. Also, an estimated 10%–20% of NSAID patients experience dyspepsia [10,11,12]. Therefore, it is justified to seek anti-inflammatory drugs from natural sources, which involve the use of bioactive natural products without or with less side effects, for the low enzymatic selectivity of NSAIDs and the abuse in their consumption that causes health problems [13].

Most nutriceuticals with major recognized biological potential as anti-inflammatory agents are triterpenes [14]. As we all know, oleanolic acid is a pentacyclic triterpene found in many medicinal plants and fruits. This chemical constituent began to be used to treat hepatitis in the 1970s, and it has shown diverse other biological effects such as antioxidant, antitumor, anti-inflammatory properties, *etc*. However, so far it has proved difficult to fully and generally apply the biological activities of oleanolic acid due to its poor water-solubility [15]. Considering the health benefits that this triterpene provides and also its limitations, in this work, we studied the oleanolic triterpenes isolated from acorns (the seeds of *Quercus serrata* var. brevipetiolata), which is a variation in the genus *Quercus* of the beech family (Fagaceae) and a kind of important deciduous tree in North and Southeast China. Appropriate anti-inflammatory indicators were selected to evaluate their anti-inflammatory activity in order to explore alternative bioactive oleanolic triterpenes.

## 2. Results and Discussion 

### 2.1. Structural Identification of Triterpenes

**Figure 1:**
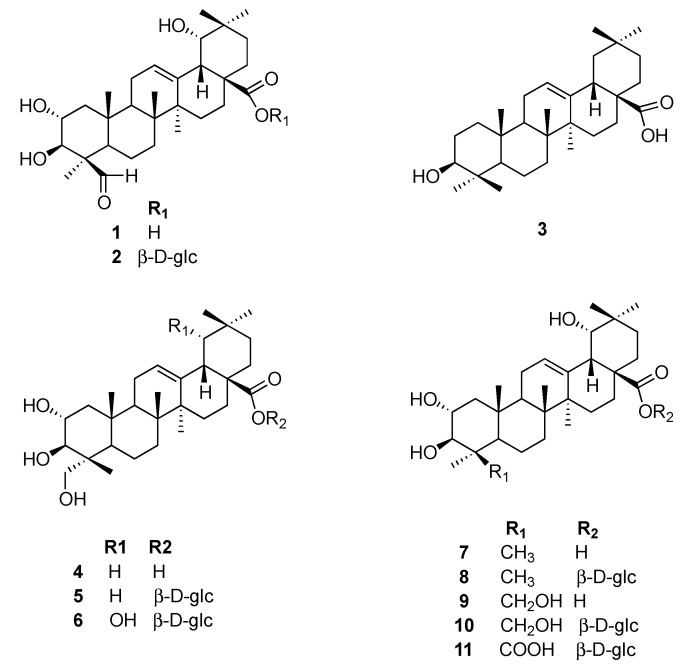
Oleanolic triterpenes isolated from acorns.

The ethyl acetate fraction of the acorn extract was separated via silica gel, Sephadex LH-20, and preparative HPLC to yield one new (compound **1**) and ten known oleanolic triterpenes **2**–**11** (Figure 1).

**Figure 2:**
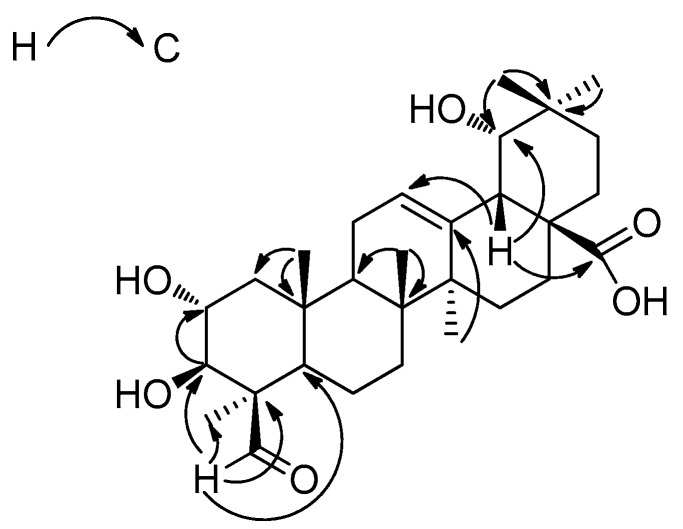
Key HMBC correlations of compound **1** (from H to C).

**Figure 3:**
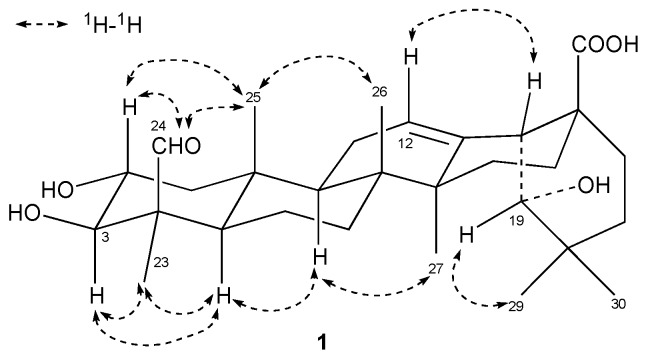
Selected NOESY correlations of compound **1**.

Compound **1** was obtained in the form of a white amorphous powder. The molecular formula was determined to be C_30_H_46_O_6_ by the HR-ESI-MS peak at *m*/*z* 503.3349 [M + H]^+^ (calcd. for C_30_H_47_O_6_, 503.3294). Its IR spectrum exhibited absorptions that corresponded to hydroxyl (3454 cm^−1^) and ester carbonyl (1697 cm^−1^) groups. The ^1^H-NMR spectrum of **1** displayed signals for six tertiary methyl groups at δ_H_ 1.64 (H-27), 1.58 (H-23), 1.21 (H-29), 1.13 (H-30), 1.01 (H-26), and 0.95 (H-25), one olefinic proton at δ_H_ 5.57 (H-12). According to the ^13^C-NMR, HMBC and DEPT spectra, 30 carbon signals were assigned, including a carboxylic carbon at δ_C_ 181.2 (C-28), one trisubstituted carbon-carbon double bond system at δ_C_ 145.3 (C-13) and 123.3 (C-12), two oxygenated methine carbons at δ_C_ 82.7 (C-3) and 68.8 (C-2), and one oxygenated methyl carbon at δ_C_ 81.6 (C-19), which suggested the presence of an olean-12-ene skeleton. The ^13^C-NMR spectrum of **1** displayed the same signals as those recorded for paradrymoniside (compound **2**) [16], except the signal at δ_C_ 181.2 (C-28), corresponding to the carbon of the esterification by a glucose, which was shifted to downfield by 3.6 ppm as a carboxylic carbon. In addition, the ^1^H-NMR spectrum displayed a signal at δ_H_ 10.45, and the ^13^C-NMR spectrum showed a peak at δ_C_ 207.7. These data were consistent with the presence of an aldehyde group. Its position on ring A was deduced by comparison of ^13^C-NMR data for **1** with those of arjunic acid (compound **7**) [17], of which the signal of C-4 (δ_C_ 41.0) was shifted to upfield by 15.3 ppm. The location of the aldehyde group at the C-24 position was deduced by the chemical shifts of C-23 and C-24, which could be compared the differentiation of the ^13^C-NMR chemical shifts between the β-methyl group (23-oxygenated triterpenes) and the α-methyl group (24-oxygenated triterpenes) [16]. Besides, the connectivity of the aldehyde group was further confirmed by the HMBC correlations. The correlations in HMBC (Figure 2) were observed between H-24 and C-3 (δ_C_ 82.7), C-23 (δ_C_ 22.3), and C-4 (δ_C_ 55.5). The ^1^H NMR spectrum also exhibited signals for two carbinol methines at δ_H_ 4.60 (1H, ddd, *J* = 12.0, 9.5, 4.6 Hz) and 3.64 (1H, d, *J* = 9.5 Hz), which were assigned to H-2β and H-3α in ring A. The relative configuration could also be confirmed by the NOESY spectrum (Figure 3), where correlations could be observed between H-2 and H-25 (β), H-3 and H-5 (α, δ_H_ 1.31), suggesting a 2α,3β-dihydroxy grouping. A 2α,3β,19α-trihydroxy moiety can also be inferred by comparison of the chemical shifts of compound **2** (δ_C_ 68.8 (C-2), δ_C_ 82.7 (C-3), 81.4 (C-19)). Moreover, a correlation between δ_H_ 10.45 and H-2 provided further confirmation of the location of the aldehyde group at C‑24. Thus, with the analysis of 2D NMR, compound **1** was elucidated to be 2α,3β,19α-trihydroxy-24-oxo-olean-12-en-28-oic acid. The proton and carbon signals are listed in Table 1.

The identities of the known compounds (Figure 1) were determined by analysis of their spectroscopic data with full assignment of their ^1^H- and ^13^C-NMR spectroscopic data and by comparison of these data with the reference values. The known compounds were identified as paradrymoniside (**2**) [16], oleanolic acid (**3**) [18], arjunolic acid (**4**) [19], arjunglucoside II (**5**) [20], arjunglucoside I (**6**) [21], arjunic acid (**7**) [17], 24-deoxysericoside (**8**) [22], sericic acid (**9**) [23], sericoside (**10**) [24], 2α,3β,19α-trihydroxyolean-12-en-24,28-dioic acid 28-β-D glucopyranoside ester (**11**) [25], respectively.

### 2.2. Anti-Inflammatory Activities

The bioactivities of the isolated oleanolic triterpenes against NO production induced by LPS in RAW 264.7 cells were examined using the Griess assay. Indomethacin was selected as positive control (IC_50_ = 47.4 μM). Except for compounds **5**, **10** and **11**, the other eight triterpenes showed potent anti-and significant inflammatory activities. Compounds **1**, **3**, **6** and **8** exhibited remarkable anti-inflammatory activities with IC_50_ values of 5.4, 7.8, 4.0 and 8.9 μM, respectively, which indicated they were more effective than indomethacin (Table 2). Meanwhile, compounds **2**, **4**, **7** and **9** also showed moderate anti-inflammatory activities, with IC_50_ values of 10.1, 13.0, 20.1 and 17.2 μM, respectively. All values were averaged from the results of three parallel experiments.

**Figure 4:**
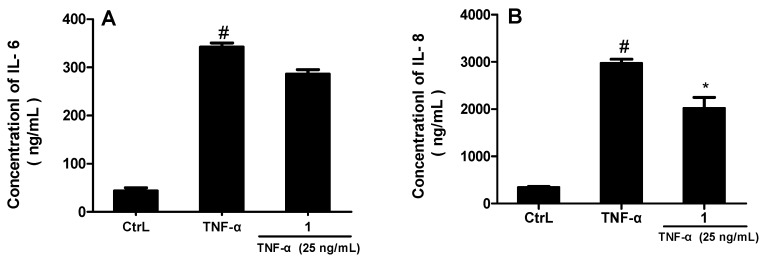
Compound **1** inhibited TNF-α-induced IL-6 and IL-8 production (* *p* < 0.05; *# p* *<* 0.01 *vs.* control).

In addition, we evaluated the effects of compound **1** on the TNFα-induced MH7A for the production of IL-6 and IL-8 by enzyme immunoassay (Figure 4). With its optimal concentrations (25 μM) determined by a preliminary screening test with the initial concentration of 20 mM, compound **1** showed different levels of inhibition of pro-inflammatory cytokines (IL-6 and IL-8). As chart A and B show, compound **1** exhibited more significant inhibition on the production of IL-8, compared with TNFα alone (*p < 0.05*).

## 3. Materials and Methods

### 3.1. General Experimental Procedures

IR spectra (KBr pellets) were recorded on a PerkinElmer 100 spectrometer (Waltham, MA, USA). NMR spectra were measured on a 500 MHz Bruker AV III spectrometer (Bruker Inc., Fällanden, Switzerland) for ^1^H, ^13^C, ^1^H-^1^H COSY, HSQC, HMBC and NOESY spectra using TMS as an internal standard. High-resolution electrospray ionization mass spectroscopic data (HR-ESI-MS) were acquired using a Waters Q-TOF Ultima mass spectrometer (Milford, MA, USA). Semi-preparative HPLC was conducted on a Waters 600 instrument equipped with a PDA detector (monitoring wavelength was set at 210 nm) and a Cosmosil 5C_8_-MS column (10ID × 250 mm, 5 μm) from Nacalai Tesque (Kyoto, Japan). Silica gel for column chromatography (300–400 mesh) was purchased from Anhui Liangchen Chemical Co. (Lu’an, China). ODS and Sephadex LH-20 gel were from Pharmacia (GE Healthcare Biosciences, Pittsburgh, PA, USA). All analytical and HPLC grade chemicals were purchased from Sinopharm Chemical Co. Ltd. (Shanghai, China). The deuterated pyridine for NMR measurements was purchased from Sigma-Aldrich, Inc. (St. Louis, MO, USA). Raw 264.7 cells were obtained by the Analysis Center of Guangdong Pharmaceutical University. MH7A cells were provided by the Shenzhen Institute of Advanced Technology. LPS and TNF-α were purchased from Sigma Chemical Co. (St. Louis, MO, USA). Greiss A and Greiss B were purchased from Aladdin Biological Technology Co., Ltd. (Shanghai, China). Mouse IL-6 and IL-8 enzyme-linked immunosorbent assay (ELISA) kits were purchased from Biolegend (San Diego, CA, USA).

### 3.2. Plant Material 

Acorns were collected in the Dabie Mountains of Anhui Province, China, in Sep. 2012, and identified as the seeds of *Quercus serrata* var. brevipetiolata by Prof. X. J. He (School of Pharmacy, Guangdong Pharmaceutical University). A voucher specimen (No. GDPU-NPR-201302) was deposited in the Lead Compound Research Institute, Guangdong Pharmaceutical University.

### 3.3. Extraction, Isolation, and Purification Procedures of Triterpenes from Acorns

Dried acorns (34.5 Kg) were extracted four times with 70% EtOH (50 L each time) under reflux, and the solvent was evaporated under vacuum to give 30 L of aqueous suspension. The suspension was partitioned successively with cyclohexane, EtOAc, and *n*-BuOH. The EtOAc extract (200 g) was subjected to silica gel column chromatography eluting in sequence with CHCl_3_/MeOH (100:1 to 0:1) to give fractions Q1 to Q18. Q7 (1.58 g) was separated on a silica gel column (petroleum ether/EtOAc at 5:1, 3:1, 1:1) to yield ten sub-fractions (Q7-1 to Q7-10). Q7-9 (231.3 mg) was applied to a silica gel column and eluted with cyclohexane/acetone at 4:1, 3:1 and 2:1 to yield four fractions (Q7-9-1 to Q7-9-4). Q7-9-3 (62.9 mg) was subjected to ODS MPLC (10 mm × 280 mm) eluted with MeOH/H_2_O (elution ratio from 30% to 100%), and purified by semi-preparative HPLC (70% MeOH/H_2_O) to get compound **1** (9.1 mg). Q10 (11.49 g) was subjected to silica gel column chromatography, eluted with chloroform/MeOH at 100:1, 80:1, 50:1, 20:1, 8:1, 5:1, 2:1, and 1:1 to yield fourteen sub-fractions. Q10-9 (929.2 mg) was chromatographed over Sephadex LH-20 (70% MeOH/H_2_O) and then purified by semi-preparative HPLC (70% MeOH/H_2_O) to obtain compounds **4** (11.2 mg) and **9** (6.0 mg). Q11 (7.04 g) was chromatographed over ODS MPLC (30 mm × 300 mm) with MeOH/H_2_O (ratio from 30% to 80%) to yield eight sub-fractions. Q11-6 (381.7 mg) was chromatographed over a Sephadex LH-20 column (CHCl_3_/MeOH, 2:1), then purified by semi-preparative HPLC (60% MeOH/H_2_O) to get compounds **2** (5.7 mg), **3** (6.7 mg), **7** (10.1 mg) and **8** (14.7 mg). Using the same ways, Q11-6 and Q11-7 (128.8 mg) were finally purified to give compounds **5** (14.0 mg) and **6** (7.0 mg). Q12 (10.1 g) was subjected to ODS MPLC (60.8 × 190 mm) with MeOH/H_2_O (ratio from 20% to 80%), then separated over Sephadex LH-20 (CHCl_3_/MeOH, 2:1) and subsequently purified by semi-preparative HPLC to yield compounds **10** (16.4 mg) and **11** (6.9 mg). All the purified compounds had been analyzed by HPLC and their purities were more than 95%.

### 3.4. Characterization of 2α,3β,19α-Trihydroxy-24-oxo-olean-12-en-28-oic acid *(**1**)*

White amorphous powder, IR (KBr) ν_max_ 3454 (OH), 2942, 1697 (C=O), 1456, 1385, 1264, 1204, 1047 cm^−1^. ^1^H-NMR (500 MHz) and ^13^C-NMR (126 MHz) data in C_5_D_5_N, see Table 1. HR-ESI-MS *m*/*z* 503.3349 [M + H]^+^ (calcd for C_30_H_47_O_6_, 503.3294).

### 3.5. Measurement of LPS-Induced Nitric Oxide (NO) Production and Cell Viability

The nitric oxide (NO) concentration in the medium was measured as an indicator of NO production according to the Griess reaction [26]. Briefly, RAW 264.7 cells were seeded into 96-well tissue culture plates at 2 × 10^5^ cells/mL and stimulated with 1 μg/mL of LPS in the presence or absence of sample. After incubation at 37 °C for 24 h, 50 μL of cell-free supernatant was mixed with 50 μL of Griess Reagent I and 50 μL of Griess Reagent II to determine nitrite production. Absorbance was measured at 546 nm against a calibration curve with sodium nitrite standard. Indomethacin was selected as a positive control. As stock solutions, all samples were dissolved in DMSO. The cell viability was examined using the MTT assay following the previously published protocols [27] to make sure the tested oleanolic triterpenes exhibited no cytotoxicity against RAW 264.7 macrophage cells at their effective concentrations. All experiments were run in triplicate (see Supplementary Materials).

### 3.6. IL-6 and IL-8 Assays

MH7A cells (2 × 10^5^ cells/mL) were incubated in the media for 24 h. Cells were then pretreated with compound **1** on its optimal concentration (25 μM) determined by a preliminary screening test with the initial concentration of 20 mM, 2 hours later followed by stimulation with 25 ng/mL of TNF-α. Following collection of culture supernatants at 6 h after TNF-α stimulation, enzyme-linked immunosorbent assay (ELISA) was performed according to the manufacturer’s protocol for quantification of levels of IL-6 and IL-8 [28,29]. All experiments were triplicated.

### 3.7. Statistical Analysis 

The results are expressed as the mean ± standard error of the mean (SEM). All the data were analyzed with one way analysis of variance (ANOVA) followed by Dunnett’s test. *p <* 0.05 was considered as statistically significant.

## 4. Conclusions

In the present study, a new oleanolic triterpene was isolated from acorns along with 10 known compounds and identified. The new compound **1** has an aldehyde group at C-24. Compounds **4** and **5** have 2α,3β,23-trihydroxy groups. Compounds **6**–**10** have 2α,3β,19α-trihydroxy moieties, and compound **11** has a carboxyl at C-24. 

From the bioactivity data, it can be concluded that most of the oleanolic triterpenes can inhibit the production of NO. Besides, comparing the results of compound **1** and **2**, **4** and **5**, **9** and **10,** the structures of aglycones have more potential activity against NO production than their glycosides. In addition, the IC_50_ values of compounds **5** and **6** were hugely different while the only difference between them is that compound **6** just has a hydroxyl more at C-19 than compound **5**, which suggests the possibility that a hydroxyl at C-19 may be an active site or can enhance the inhibitory effect against NO production. In addition, the results of IL-6 and IL-8 assays also implied that compound **1** may inhibit the release of inflammatory cytokines to exert anti-inflammatory effects.

In brief, the adverse effects of nonsteroidal anti-inflammatory drugs (NSAIDs) have become increasingly common, and it is urgent to find new anti-inflammatory agents like the oleanolic triterpenes. The oleanolic triterpenes from acorns appeared to contribute to the anti-inflammatory properties of the extract, and are beneficial for human health. However, with the limited data available it is still difficult to make more detailed conclusions about certain positions which may play a part in the anti-inflammatory activity and how these compounds work. Because of the potential importance and value of these oleanolic triterpenes with effective anti-inflammatory activity, more experiments on other more inflammatory factors should be performed to study their mechanism of action, and more studies should also be done to unfold other hidden aspects of the components of acorns.

## Figures and Tables

**Table 1:** ^1^H- and ^13^C-NMR Spectroscopic Data for Compound **1** ^a^ in C_5_D_5_N.

Position	Compound 1	
	δ_H_	δ_C_
1	1.35, m; 2.33, ddd (12.0, 9.5, 4.6)	47.2
2	4.60, td (11.3, 4.6)	68.8
3	3.64, d (9.5)	82.7
4		55.5
5	1.31	57.7
6	1.50, d (9.3); 1.87, d (8.0)	20.0
7	1.33, m; 1.47, m	33.5
8		40.2
9	1.99, s	47.5
10		39.0
11	2.02, m; 2.15, m	25.1
12	5.56, t-like	123.3 ^a^
13		145.3
14		42.6
15	2.13–2.11, m	29.4
16	2.86–2.78, m	28.7
17		46.4
18	3.62, brs	45.2
19	3.63, m ^b^	81.6
20		36.1
21	1.13, m; 1.22, m	29.5
22	2.12, m; 2.19, m	33.5
23	1.58, s	22.3
24	10.45, s	207.7
25	0.95, s	17.6
26	1.01, s	17.7
27	1.64, s	25.1
28		181.2 ^a^
29	1.21, s	29.1
30	1.13, s	25.2

^1^H (δ ppm, *J* in Hz, s: singlet; d: doublet; t: triplet; brs: broad singlet; m: multiplet); ^1^H-NMR data assignments are based on the HSQC and HMBC. ^a^ Overlapped signals were assigned from HSQC and HMBC. CH_3_, CH_2_, CH, and C multiplicities were determined by DEPT 135°. ^b^ Signal overlapped with H-3 and H-18.

**Table 2:** Inhibitory Effects on NO Production of the Triterpenes from Acorns.

Compound	IC_50_ (μM)	Compound	IC_50_ (μM)
**1**	5.4 ± 1.2	**7**	20.1 ± 6.4
**2**	10.1 ± 0.8	**8**	8.9 ± 0.7
**3**	7.8 ± 2.4	**9**	17.2 ± 0.7
**4**	13.0 ± 0.2	**10**	>100
**5**	>100	**11**	>100
**6**	4.0 ± 0.8	Indomethacin ^a^	47.4 ± 4.5

^a^ positive control.

## Supplementary Materials

Supplementary materials can be accessed at: http://www.mdpi.com/1420-3049/21/5/669/s1.
