# Supplementary material for: Anti-Inflammatory Oleanolic Triterpenes from Chinese Acorns
Source: Molecules. 2016 May 20;21(5):669. doi: 10.3390/molecules21050669 (PMC6272836; doi:10.3390/molecules21050669)
Supplement: Supplementary file 1 [file molecules-21-00669-s001.pdf]

Supplementary Materials: Anti-Inflammatory Oleanolic Triterpenes from Chinese Acorns

Jie Huang, Yihai Wang, Chuan Li, Xinluan Wang and Xiangjiu He

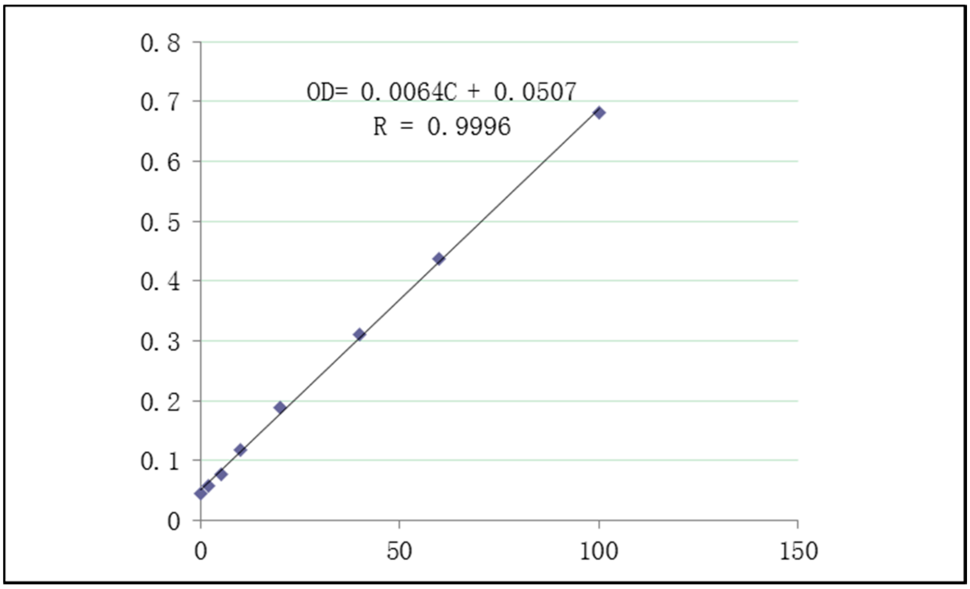

Figure S1. The Standard Curve of the NO Assay. C: the concentrations of $NaNO_2$ (μM); OD: the absorbance value.

The standard curve of NO assay were tested by the Nitric oxide kits. NO concentration was photometrically quantified for the amounts of stable product nitrite produced in the cell using the nitrate reduction method. So the X axis means the concentrations of NaNO2 (μM), the Y axis means the absorbance values. IC50 values were the OD values of each compounds calculated according to the standard curve of NO with a calculation software CalcuSyn.

NO inhibition rate = (OD value average of model control group − OD value average of the sample group)/(OD value average of model control group − OD value average of negative control group) × 100%

The Results of the MTT Assay

Table S1. The Average OD Values of Compounds **1–11**.

Compound	OD of 100 μM	OD of 50 μM	OD of 25 μM
Control group	2.50	2.36	2.21
**1**	2.27	2.44	2.40
**2**	2.26	2.43	2.32
**3**	2.53	2.33	2.34
**4**	2.04	2.28	2.35
**5**	2.35	2.43	2.50
**6**	2.18	2.40	2.27
**7**	2.27	2.34	2.50
**8**	2.40	2.29	2.19
**9**	1.97	2.27	2.18
**10**	2.40	2.29	2.25
**11**	2.24	2.34	2.00

OD: the average absorbance value.

Table S2. The Inhibition Ratios of Compounds.

Compound	100 µM(%)	50 µM(%)	25 µM(%)
**1**	9.20	−3.39	−8.60
**2**	9.60	−2.97	−4.98
**3**	−1.20	1.27	−5.88
**4**	18.40	3.39	−6.33
**5**	6.00	−2.97	−13.12
**6**	12.80	−1.69	−2.71
**7**	9.20	0.85	−13.12
**8**	4.00	2.97	0.90
**9**	21.20	3.81	1.36
**10**	4.00	2.97	−1.81
**11**	10.40	0.85	9.50

From Table S1, we can see the average OD values of compounds **1–11**, and the data were calculated by three parallel groups of every concerntration of each compound.

From Table S2, the inhibition ratios were resulted from the following formula:

The inhibition ratios = [(OD of control group) − (OD of each compound)]/(OD of control group) × 100%

And from the results we can see, all the inhibition ratios were less than 50%, which means compounds **1–11** exhibited no cytotoxicity against the tested cells (RAW 264.7 macrophage cells).

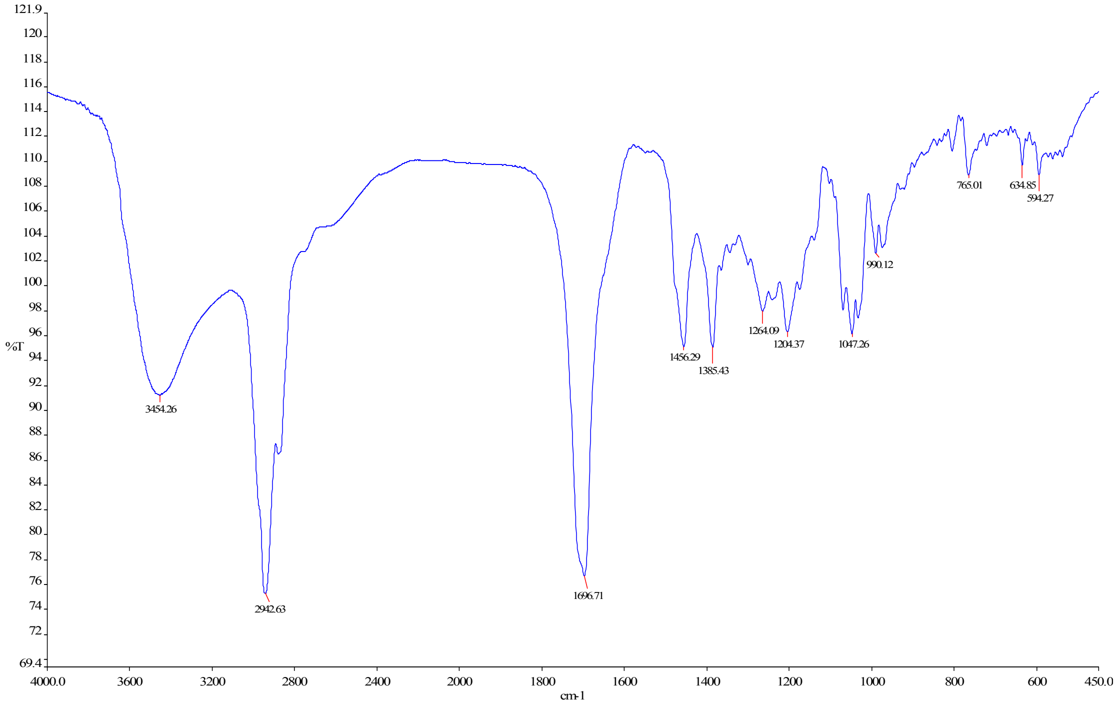

Figure S2. IR of compound **1**.

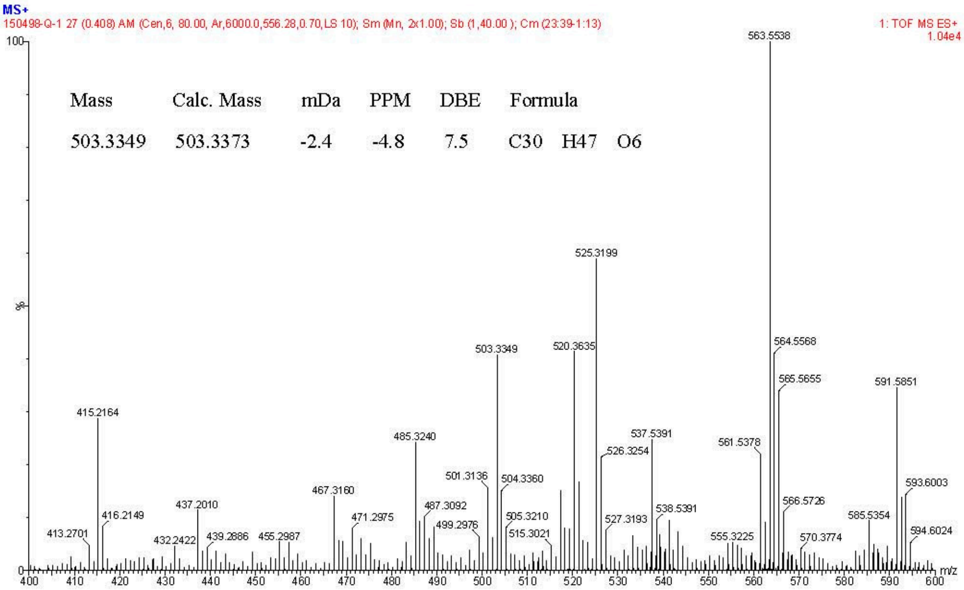

Figure S3. HR-ESI-MS of Compound **1**.

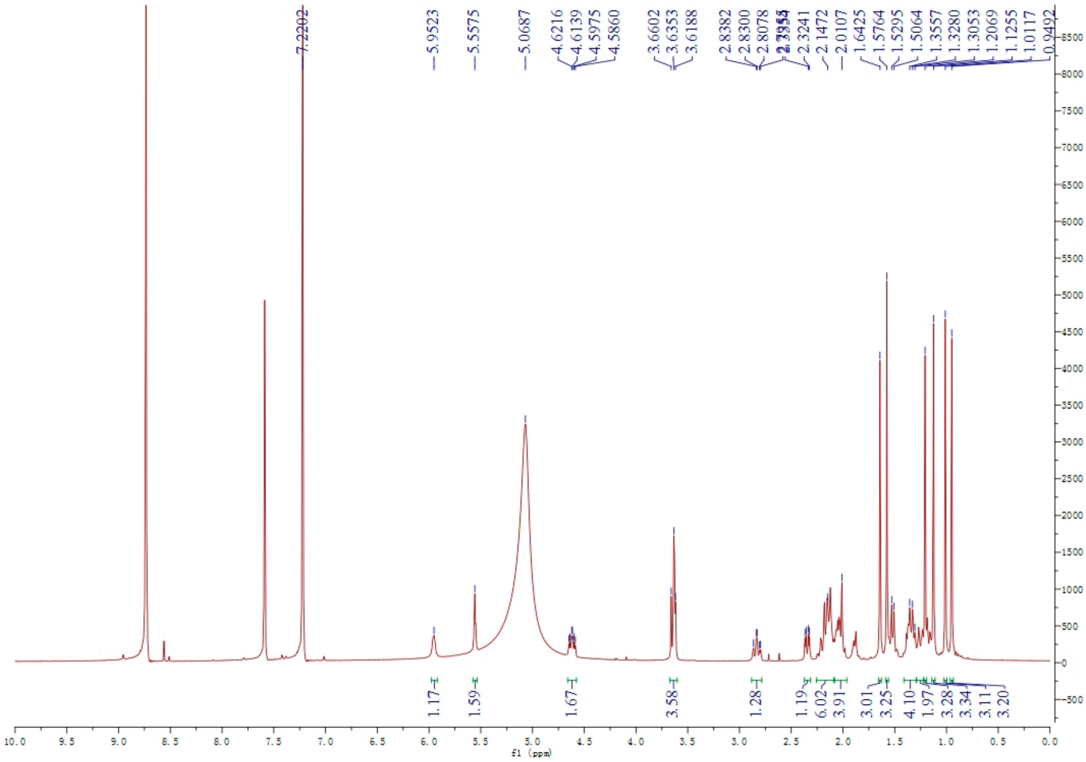

Figure S4. ^{1}H-NMR of Compound **1** (500 MHz, C5D5N).

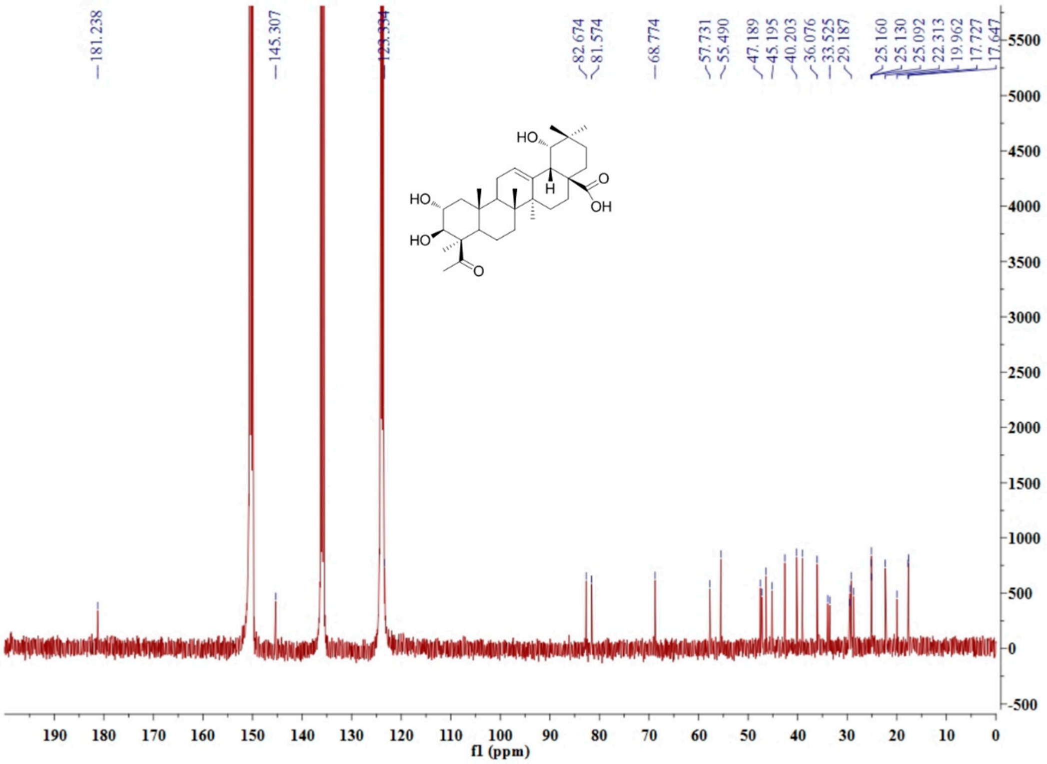

Figure S5. ^{13}C-NMR of Compound **1** (101 MHz, C5D5N).

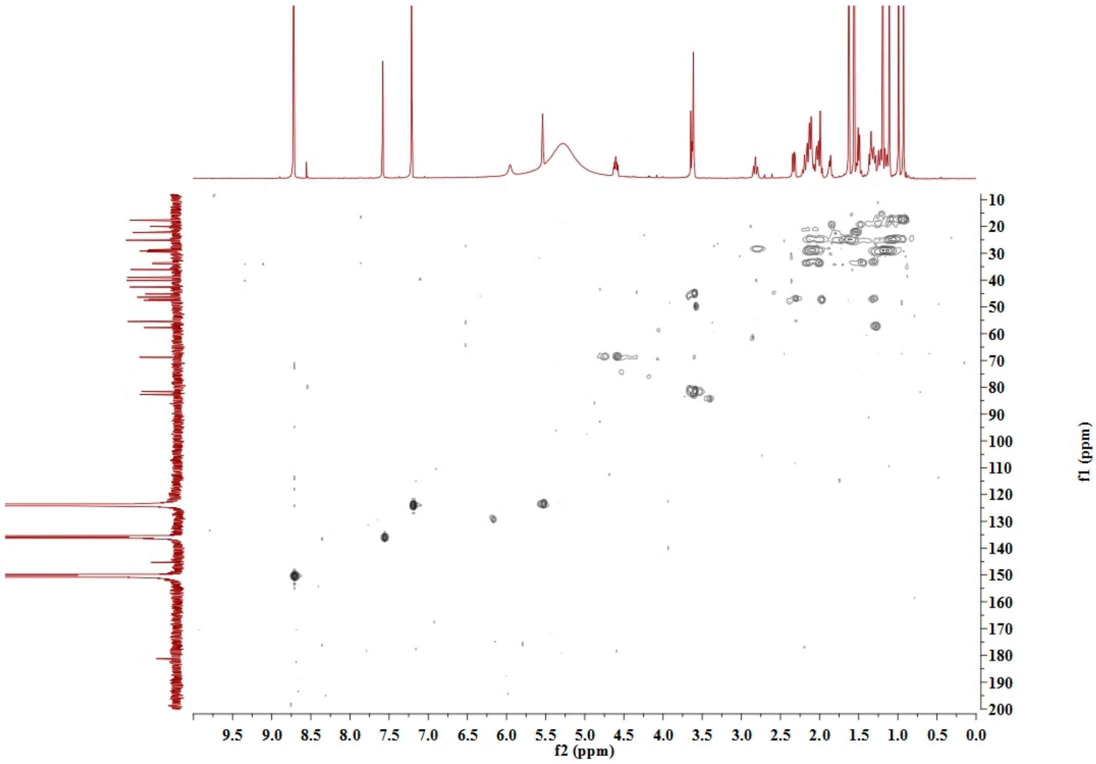

Figure S6. HSQC of Compound **1**.

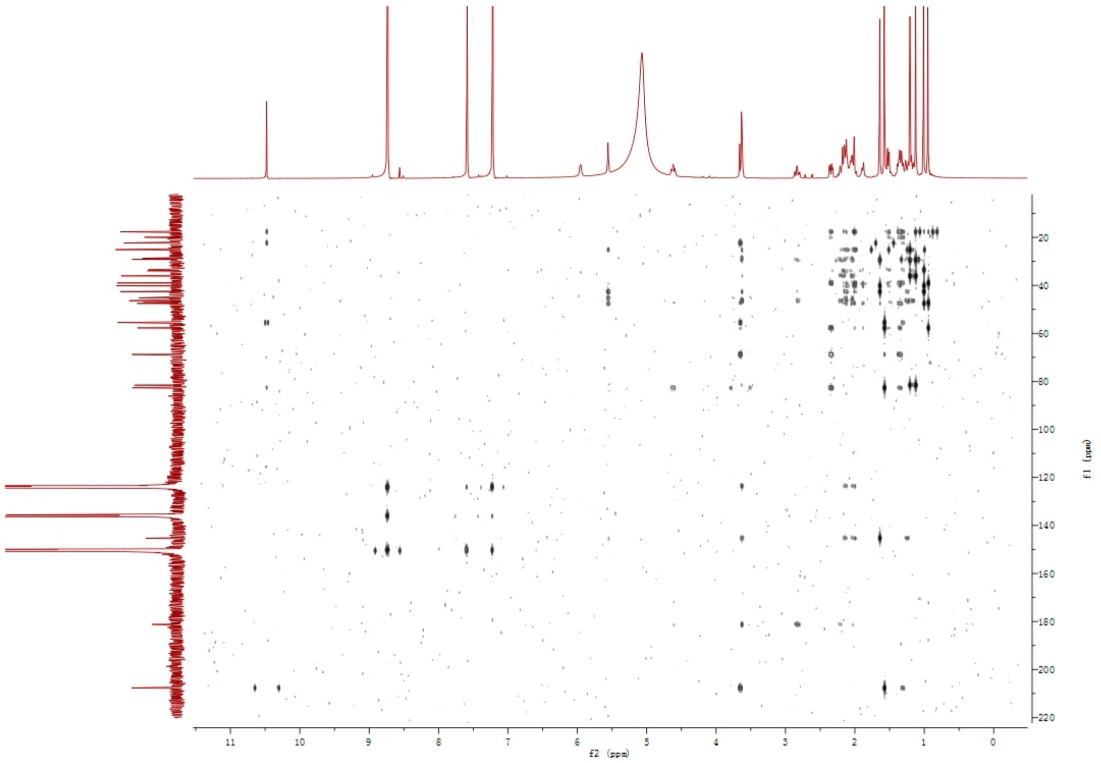

Figure S7. HMBC of Compound **1**.

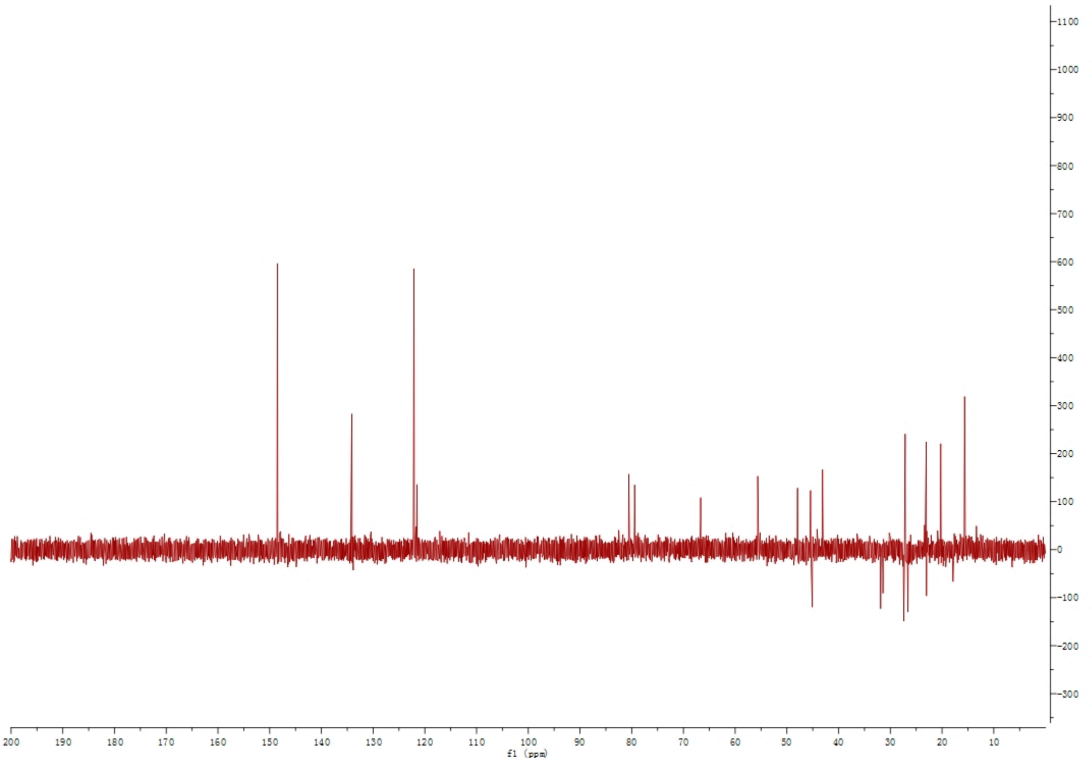

Figure S8. DEPT 135°of Compound **1**.

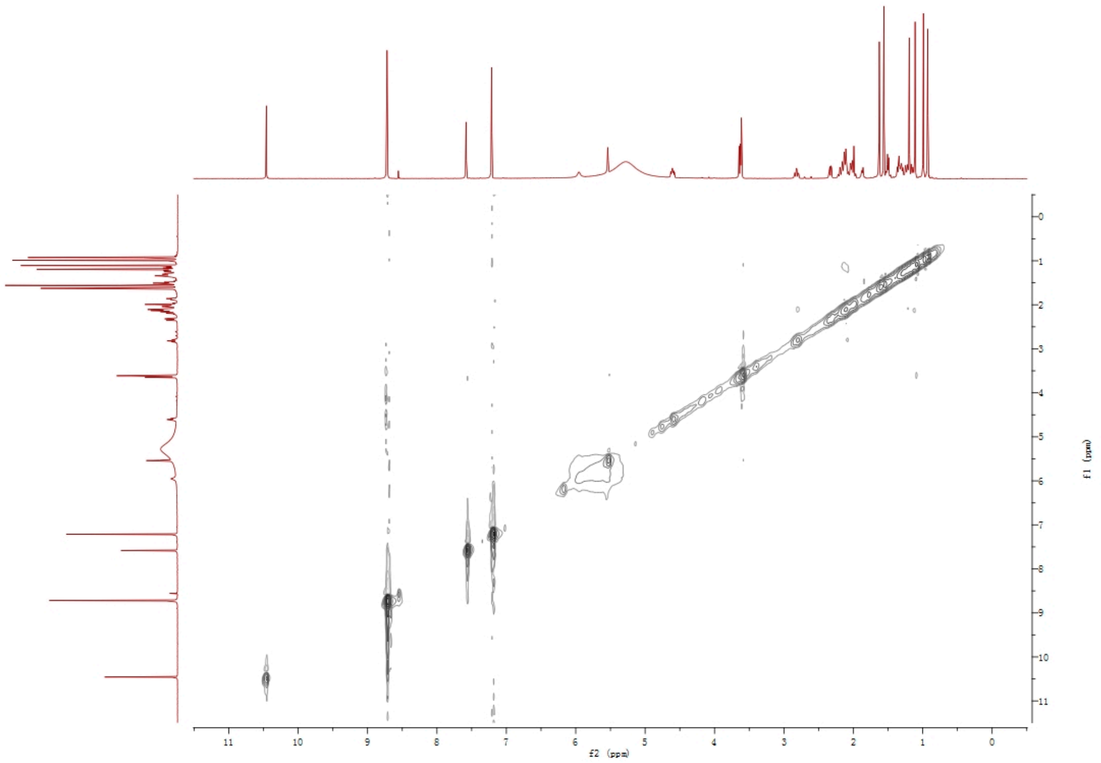

Figure S9. NOESY of Compound **1**.

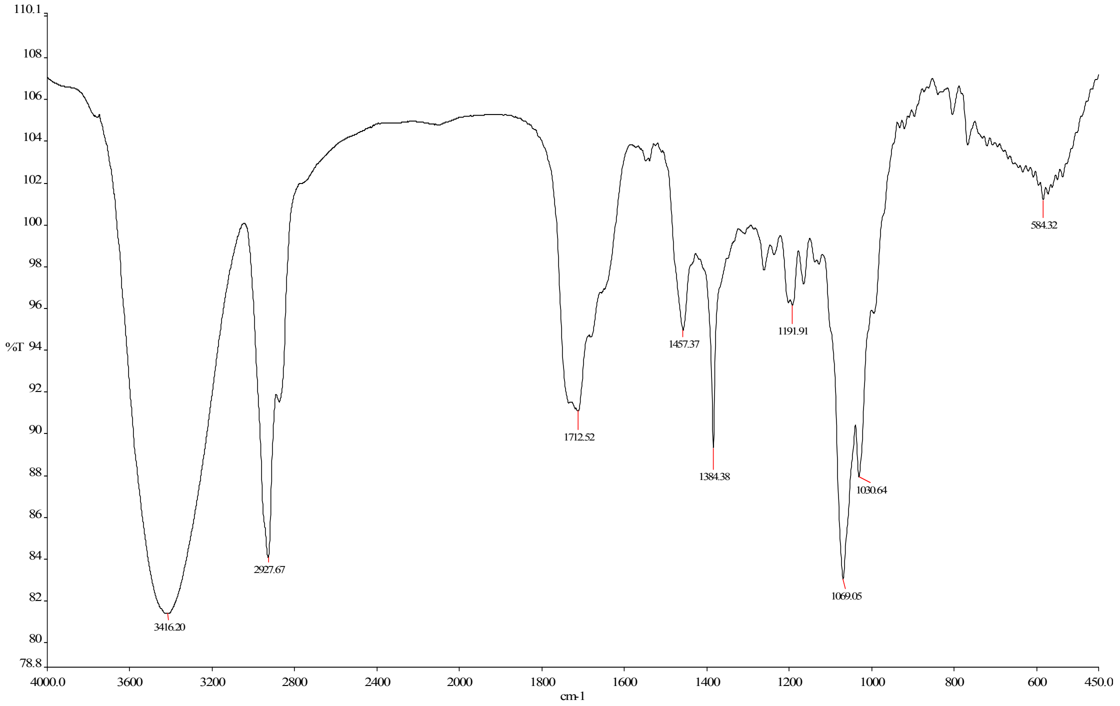

Figure S10. IR of Compound **2**.

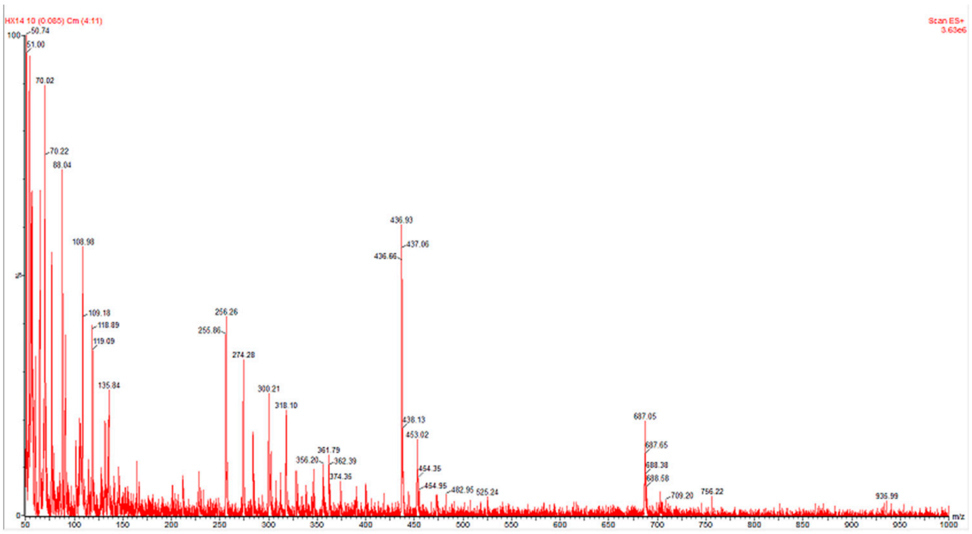

Figure S11. ES-MS of Compound **2**.

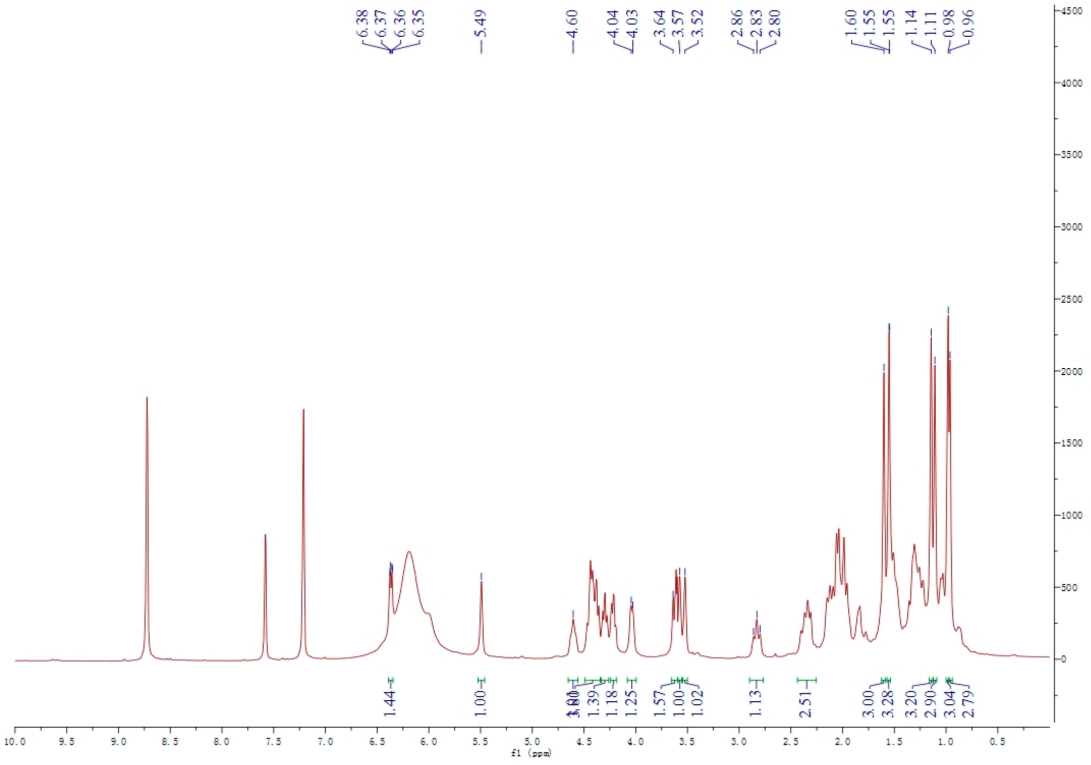

Figure S12. ^{1}H-NMR of Compound **2** (500 MHz, C5D5N).

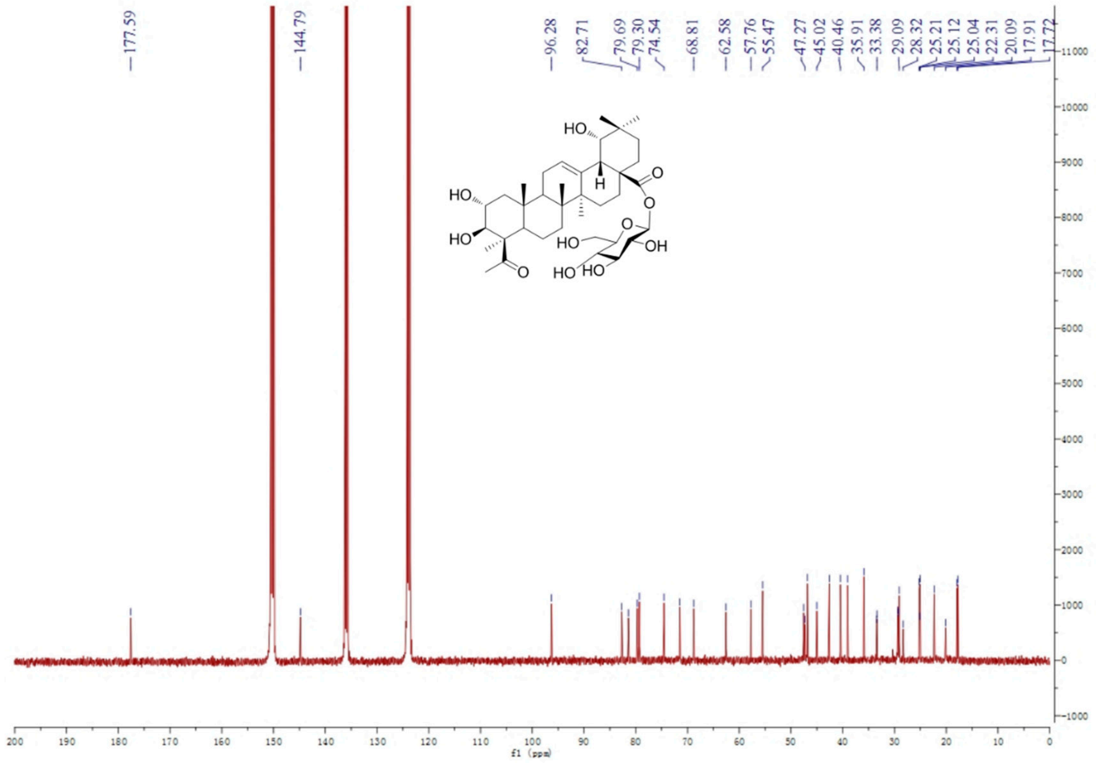

Figure S13. ^{13}C-NMR of Compound **2** (126 MHz, C5D5N).

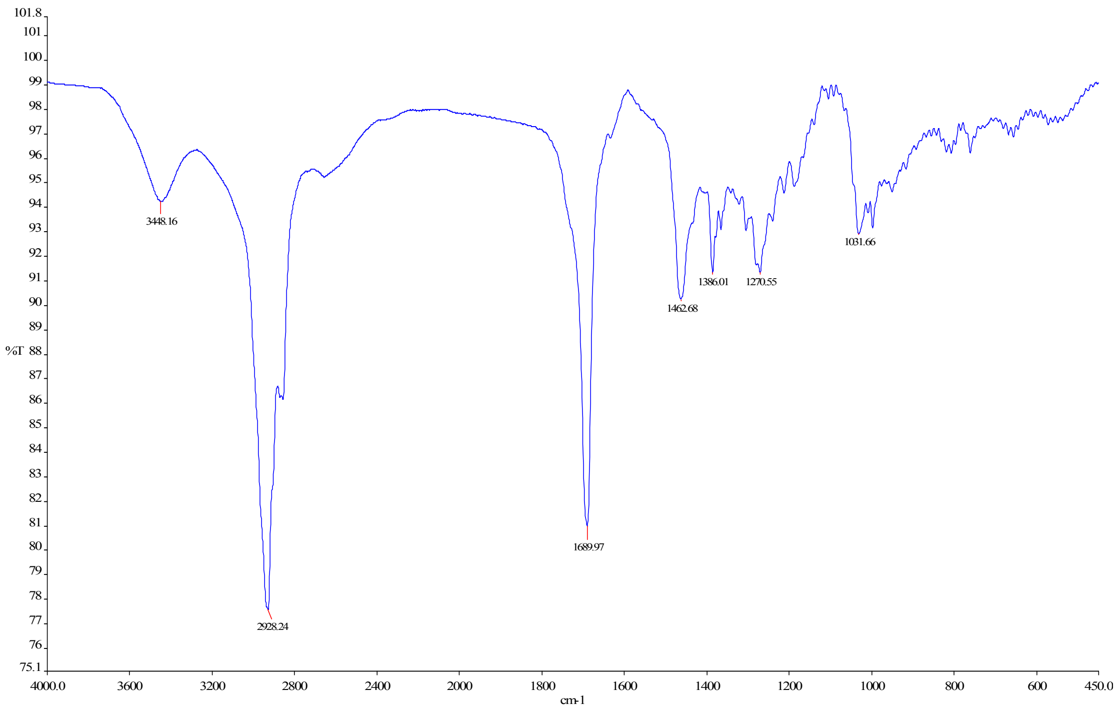

Figure S14. IR of Compound **3**.

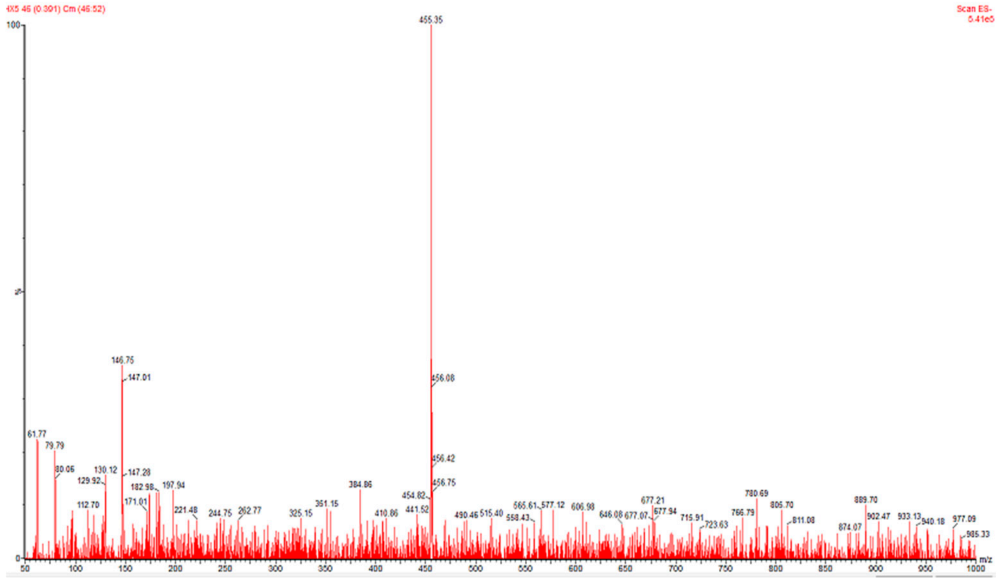

Figure S15. ES-MS of Compound **3**.

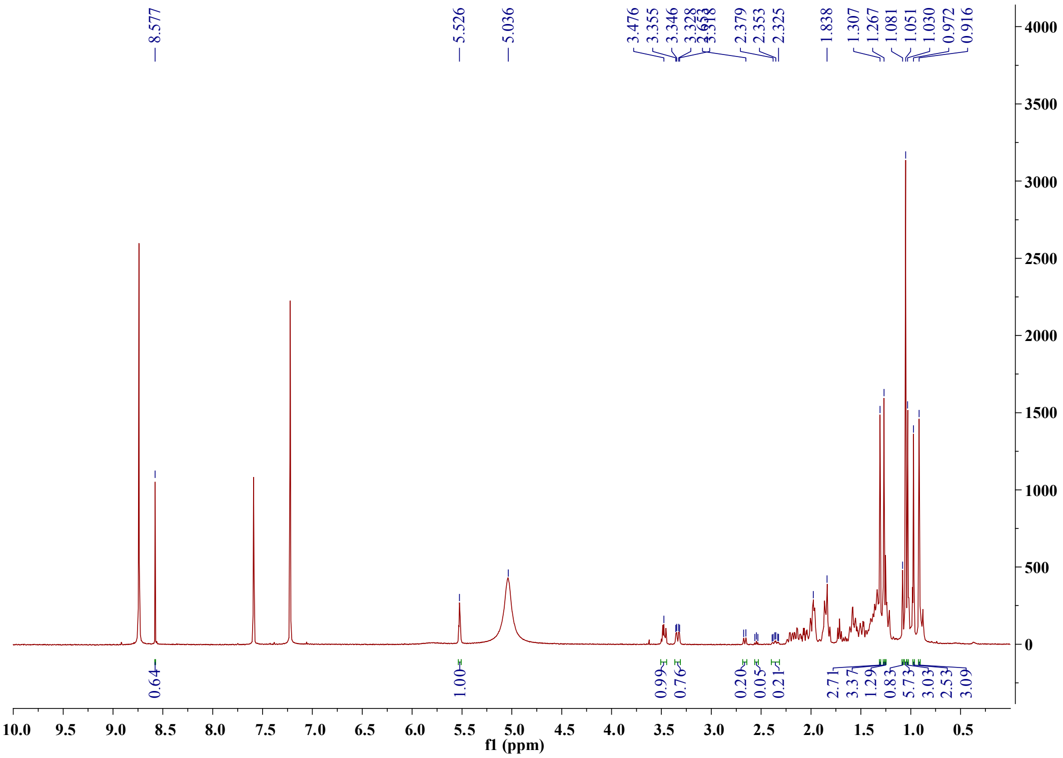

Figure S16. ^{1}H-NMR of Compound 3 (500 MHz, C5D5N).

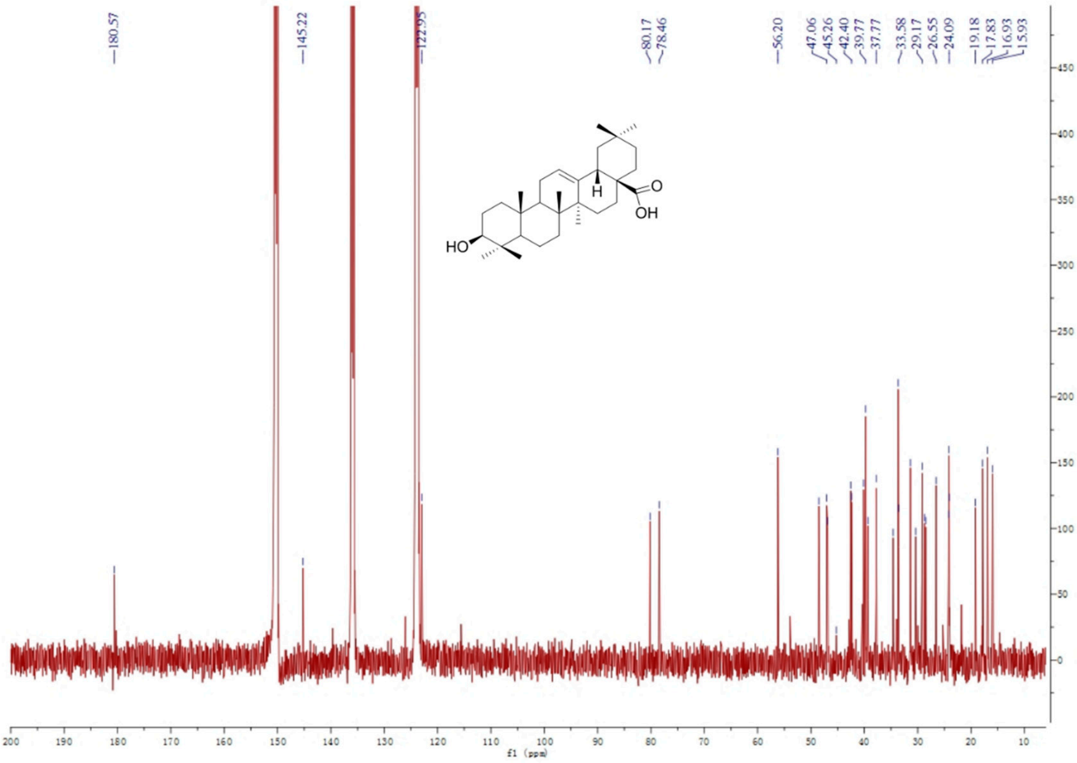

Figure S17. ^{13}C-NMR of Compound **3** (126 MHz, C5D5N).

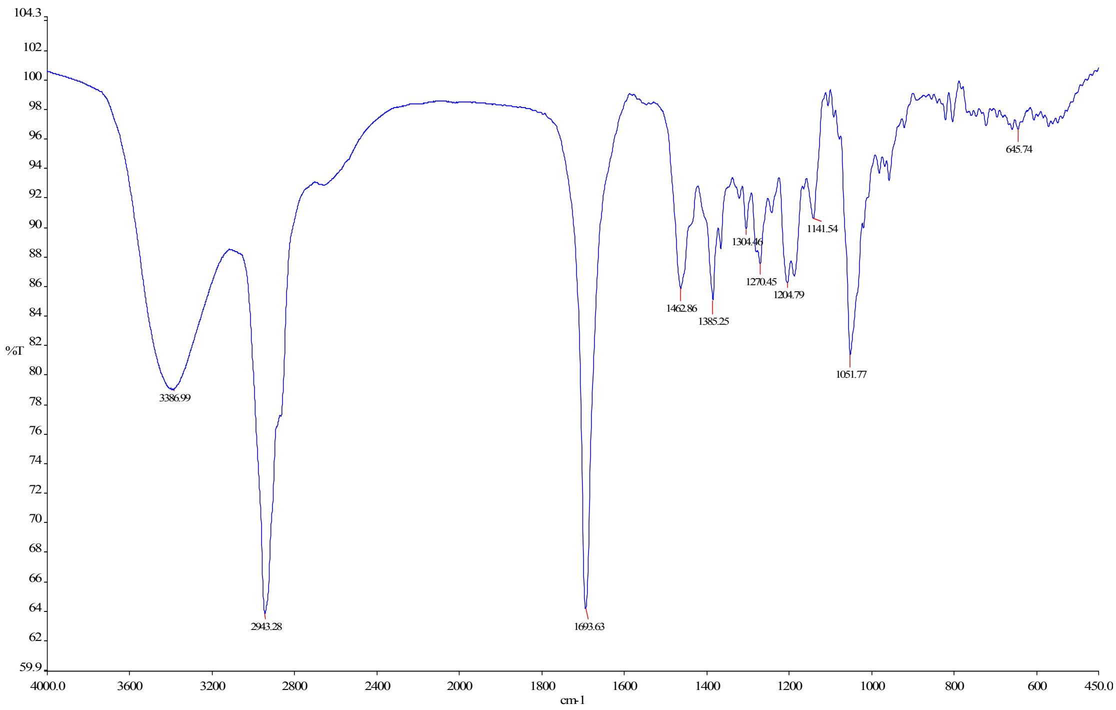

Figure S18. IR of Compound **4**.

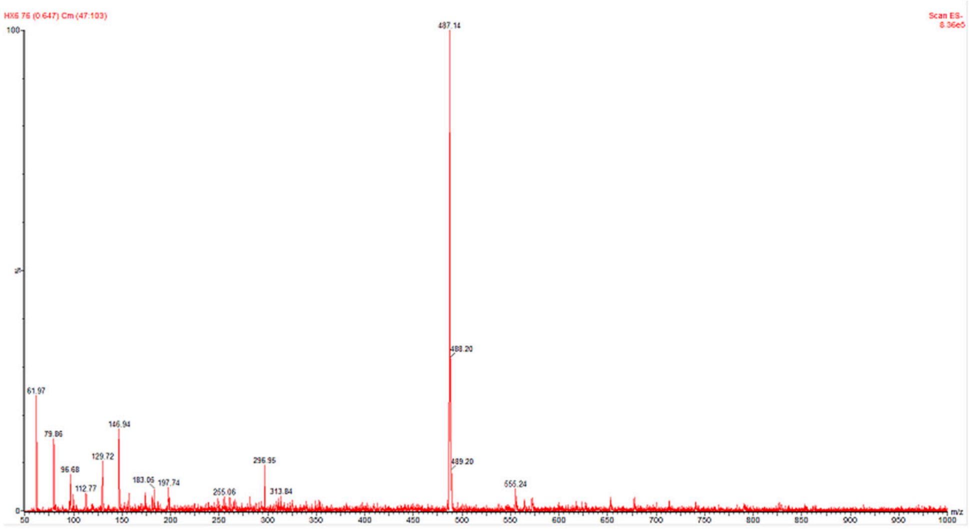

Figure S19. ES-MS of Compound **4**.

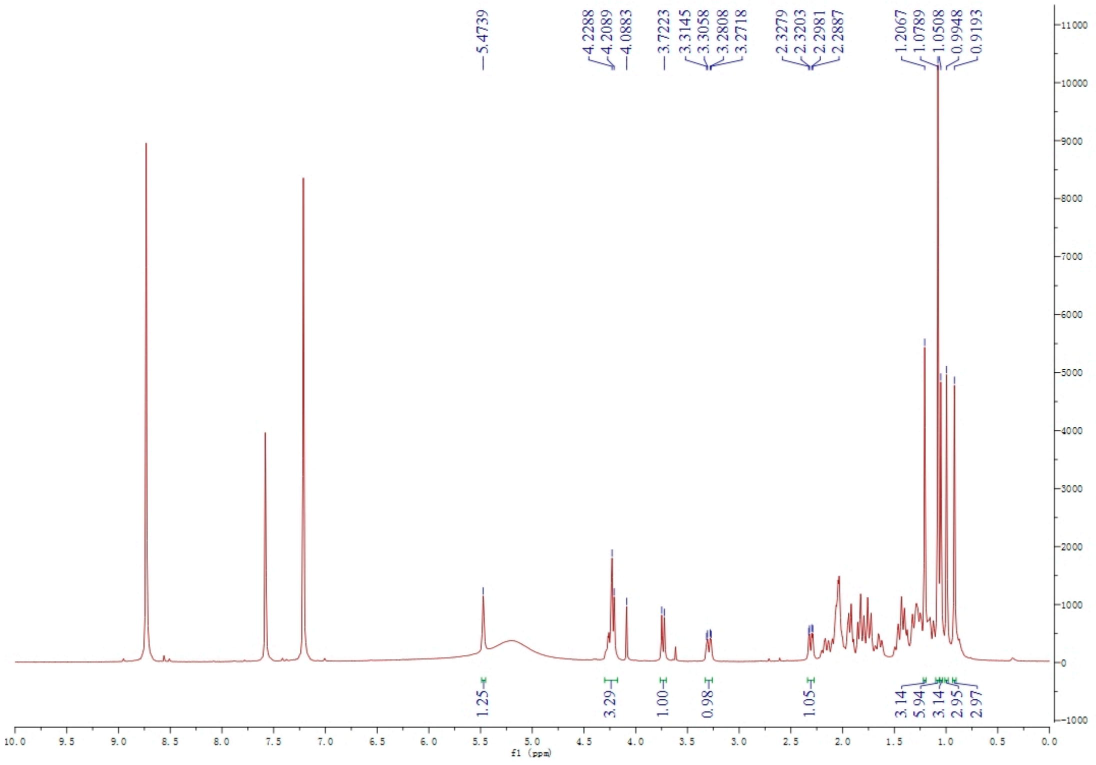

Figure S20. ^{1}H-NMR of Compound **4** (500 MHz, C5D5N).

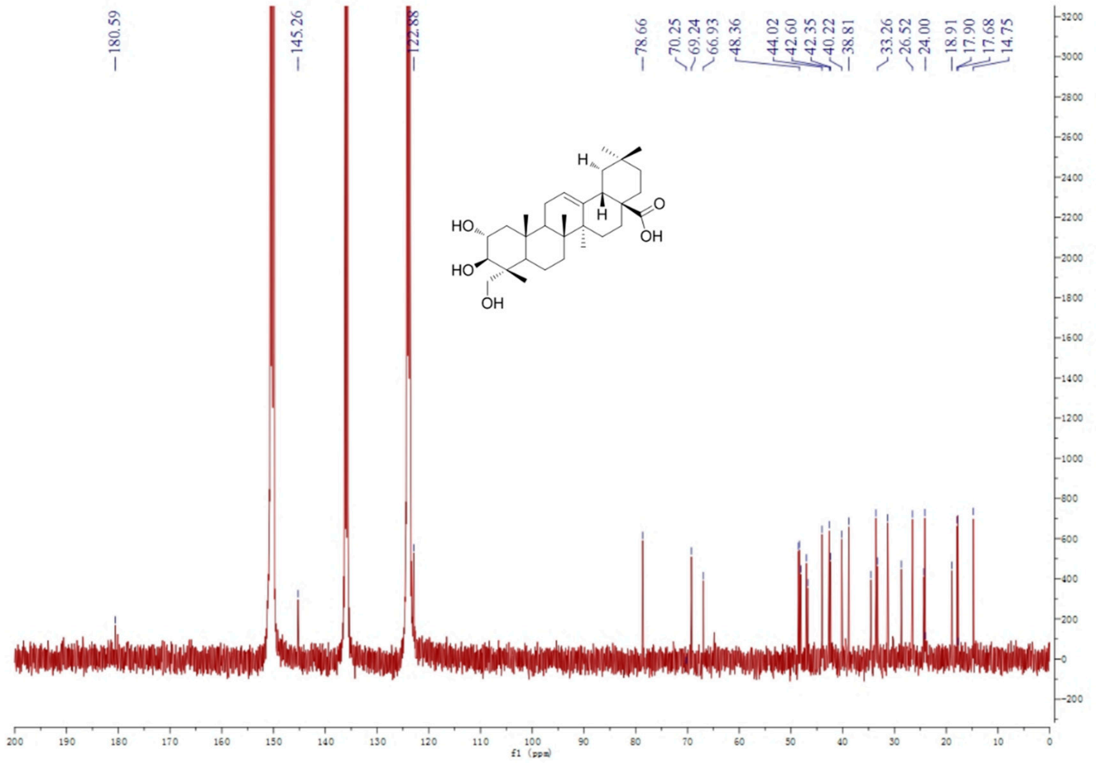

Figure S21. ^{13}C-NMR of Compound **4** (126 MHz, C5D5N).

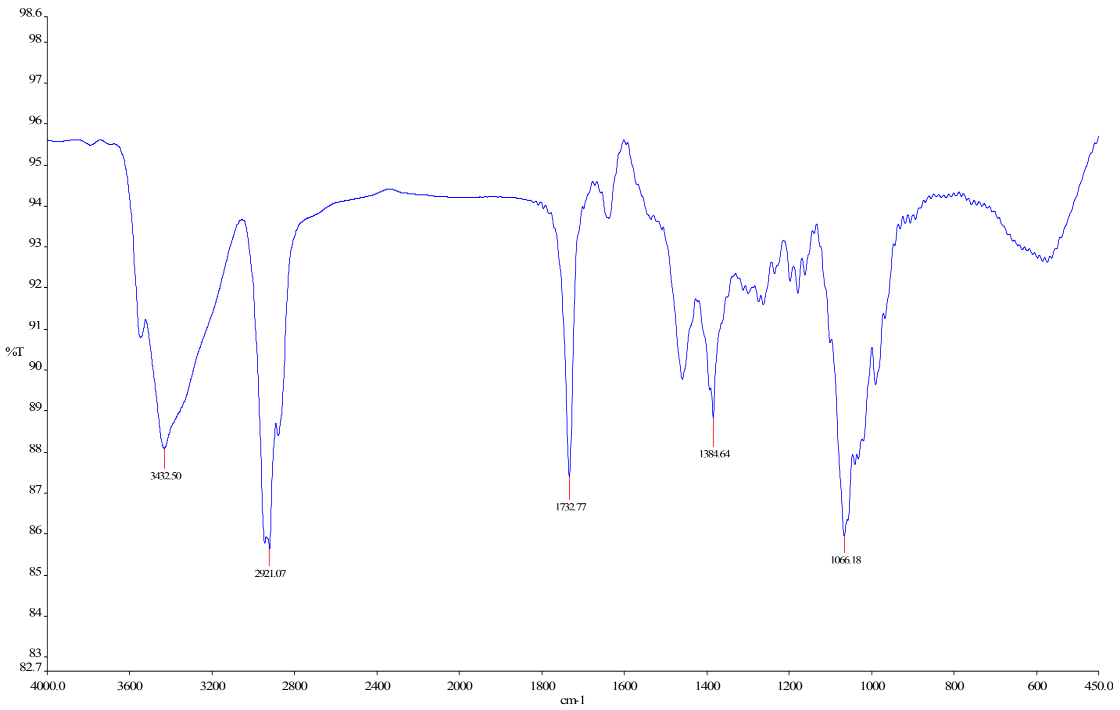

Figure S22. IR of Compound **5**.

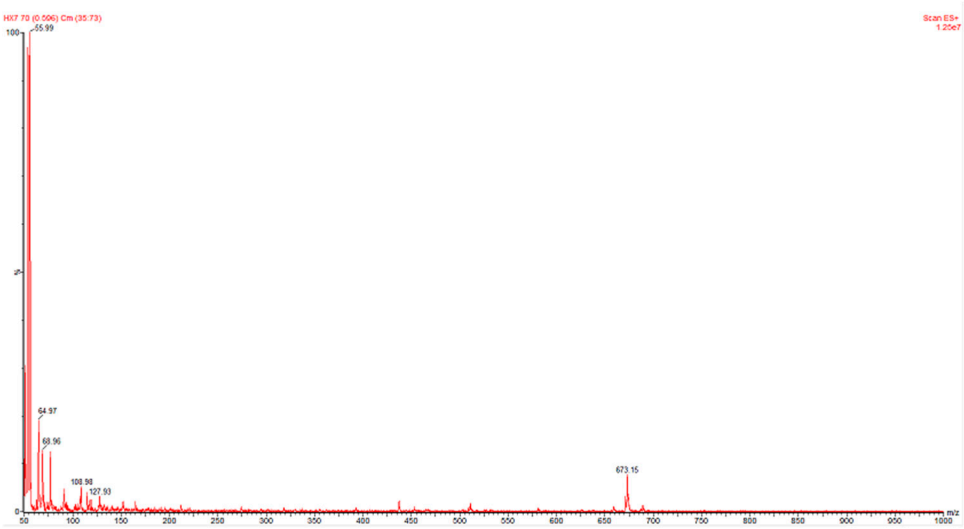

Figure S23. ES-MS of Compound **5**.

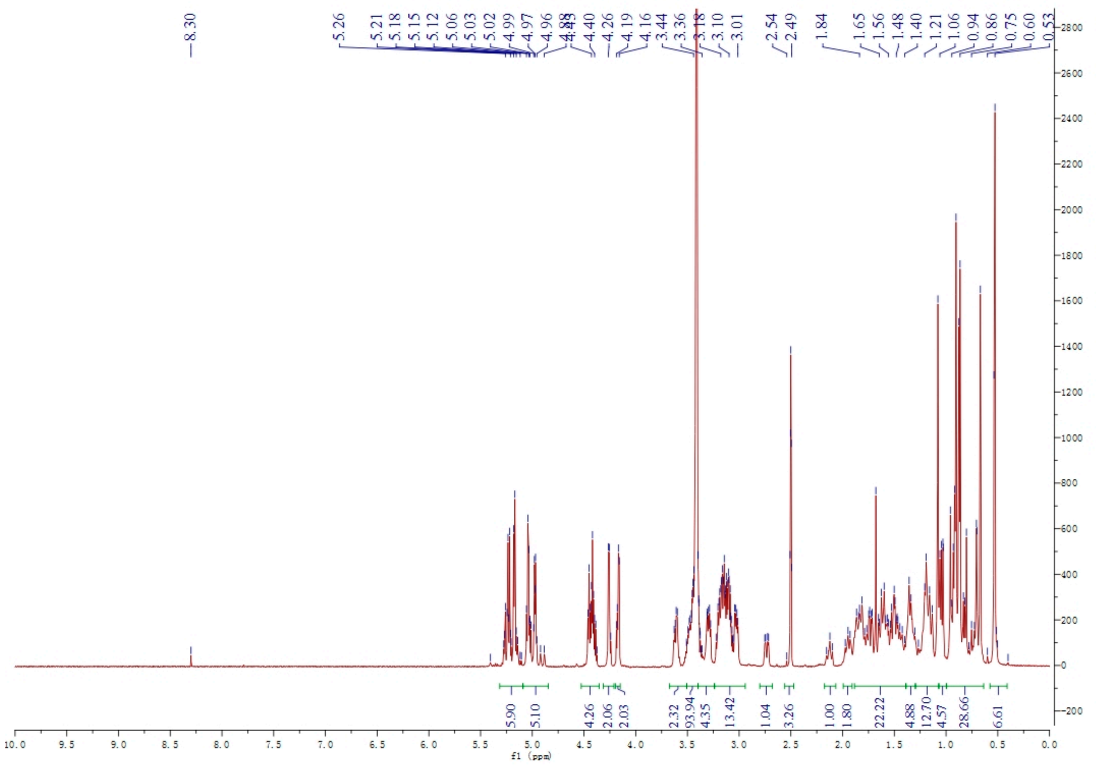

Figure S24. ^{1}H-NMR of Compound **5** (500 MHz, DMSO).

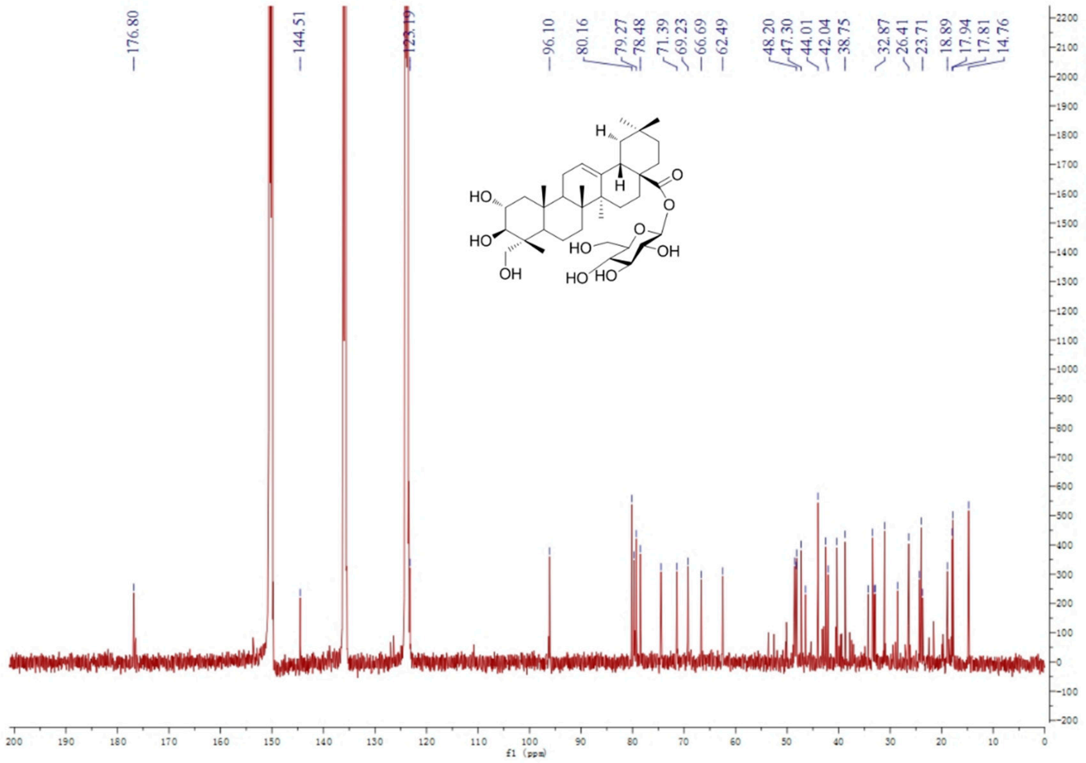

Figure S25. ^{13}C-NMR of Compound **5** (126 MHz, DMSO).

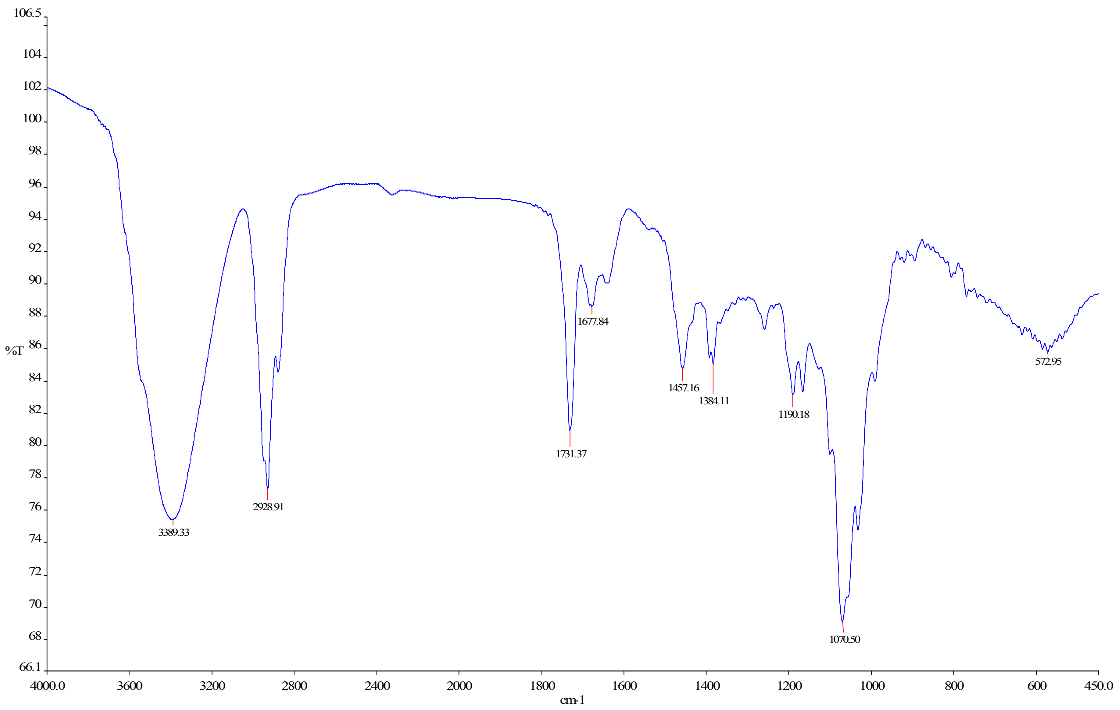

Figure S26. IR of Compound **6**.

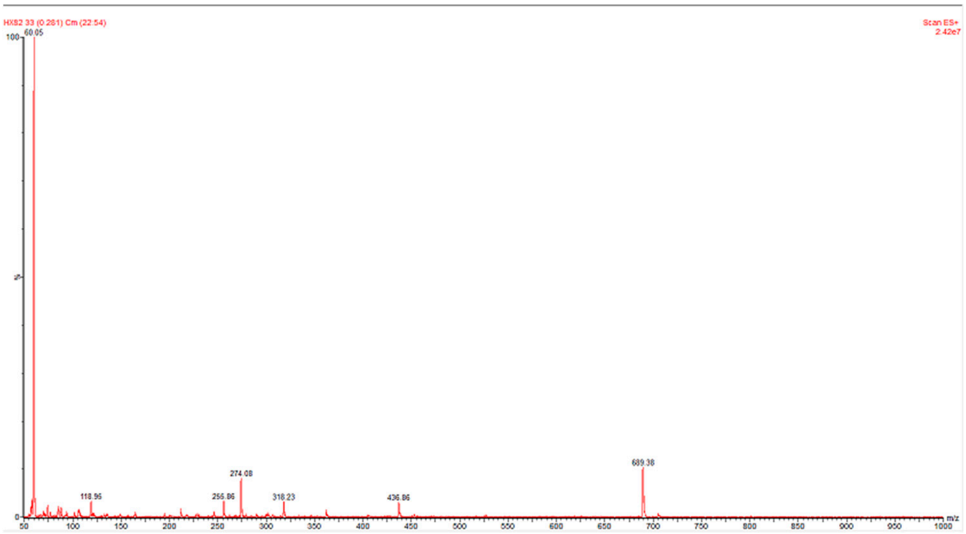

Figure S27. ES-MS of Compound **6**.

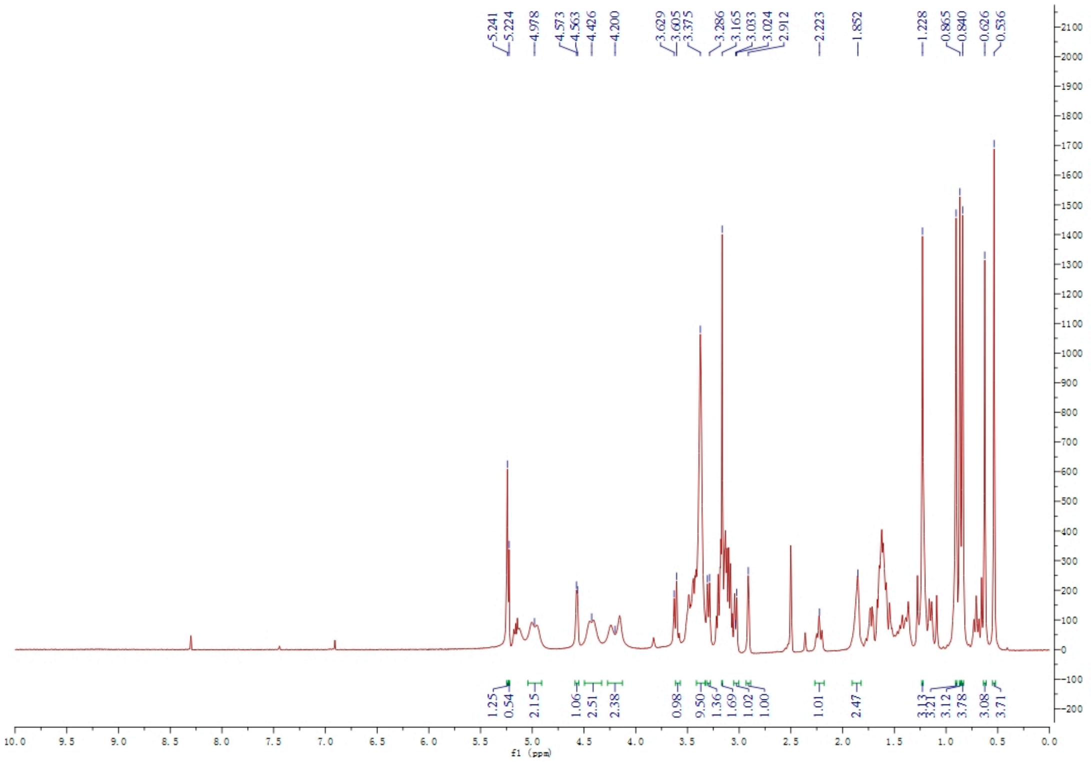

Figure S28. ^{1}H-NMR of Compound **6** (500 MHz, DMSO).

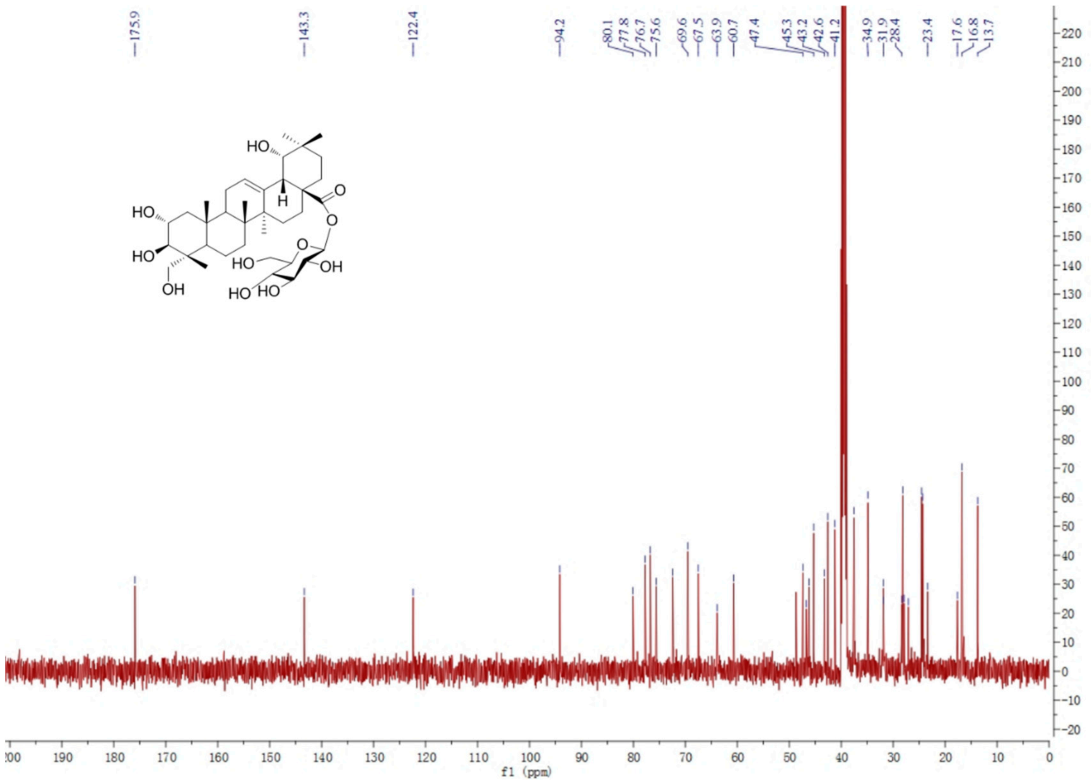

Figure S29. 13C-NMR of Compound **6** (126 MHz, DMSO).

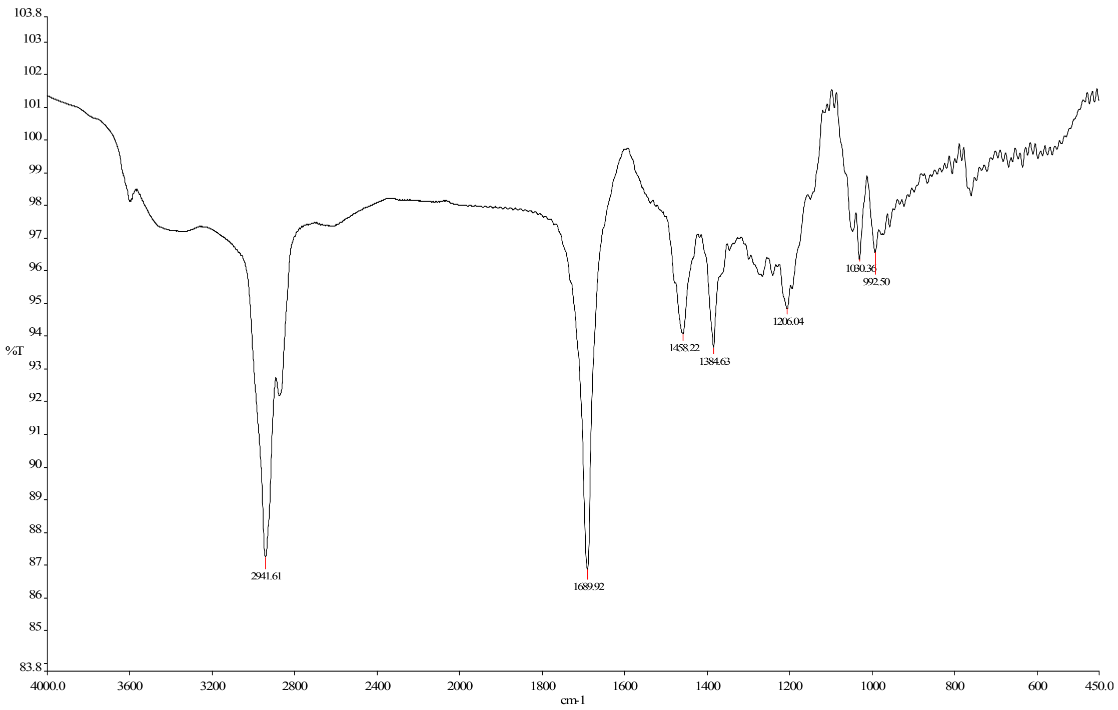

Figure S30. IR of Compound **7**.

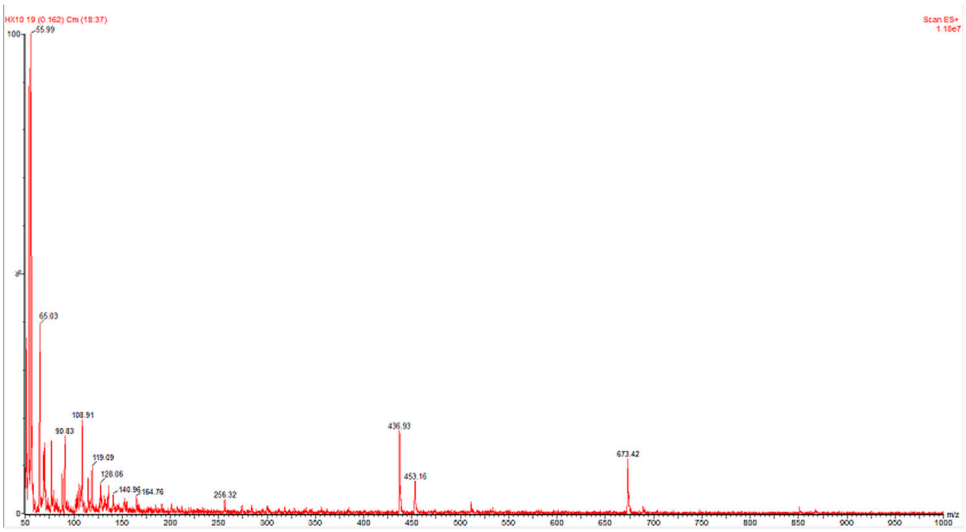

Figure S31. ES-MS of Compound **7**.

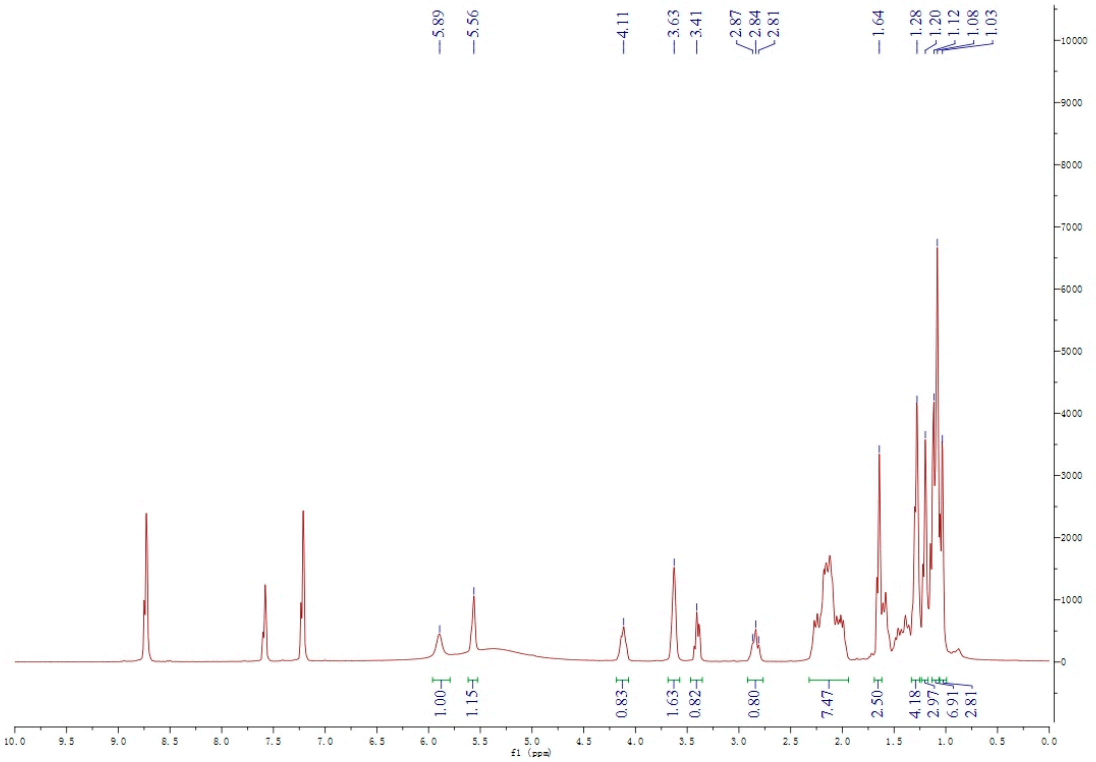

Figure S32. ^{1}H-NMR of Compound 7 (400 MHz, C5D5N).

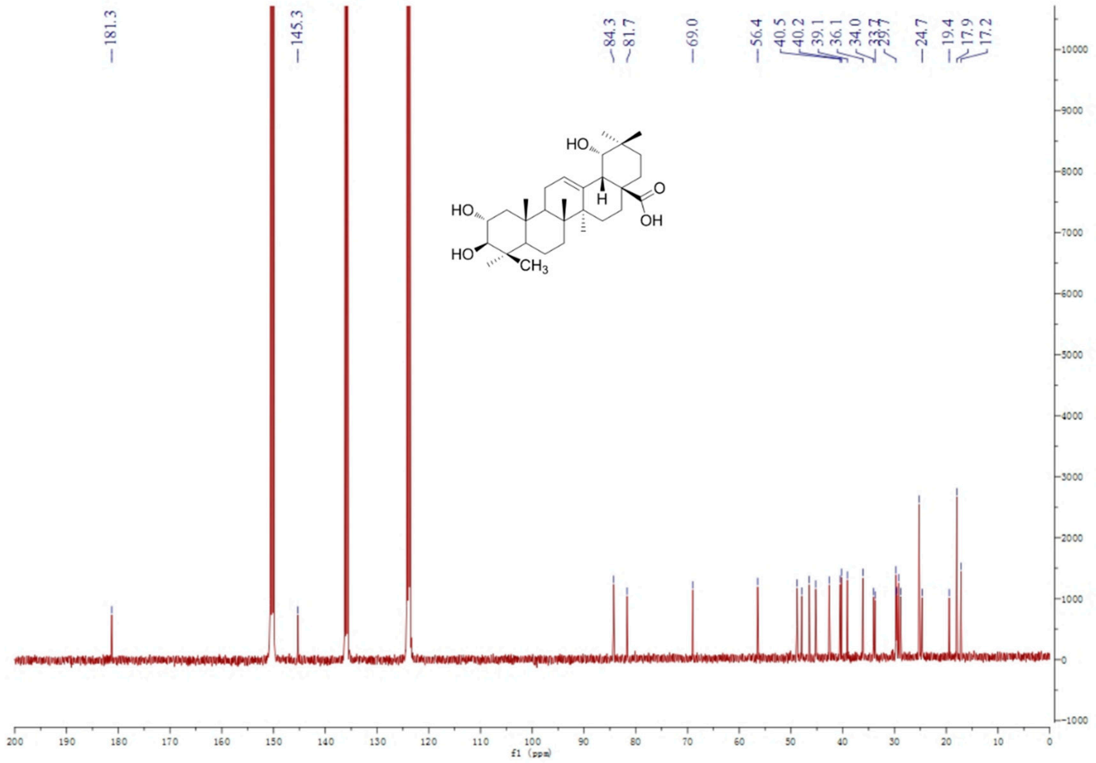

Figure S33. ^{13}C-NMR of Compound **7** (101 MHz, C5D5N).

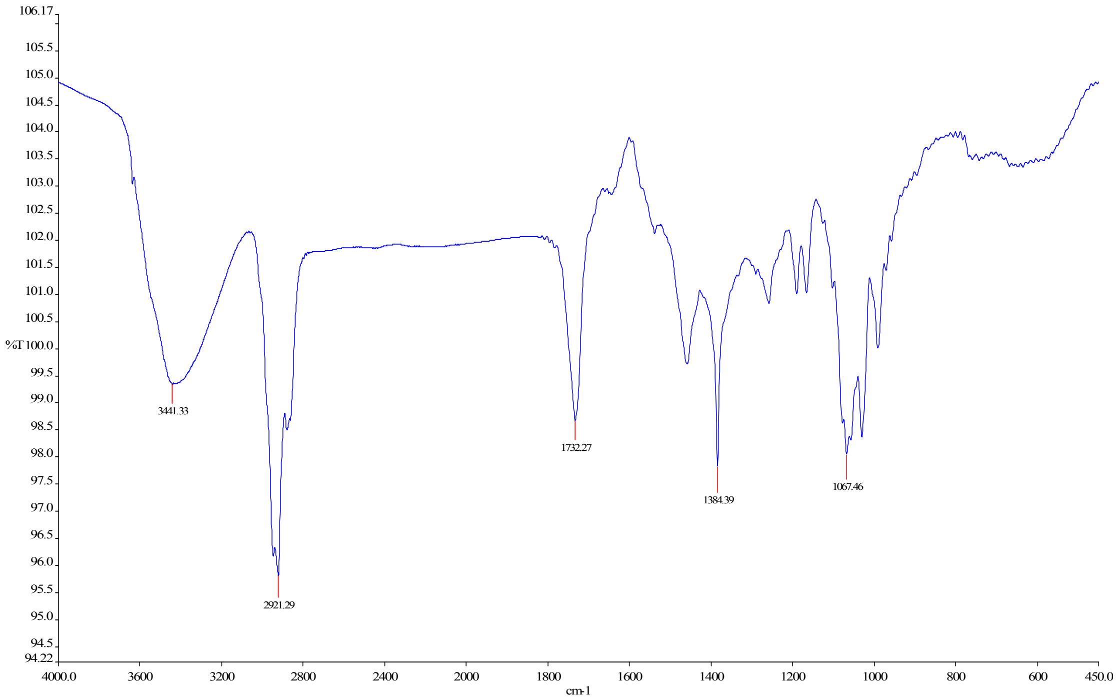

Figure S34. IR of Compound **8**.

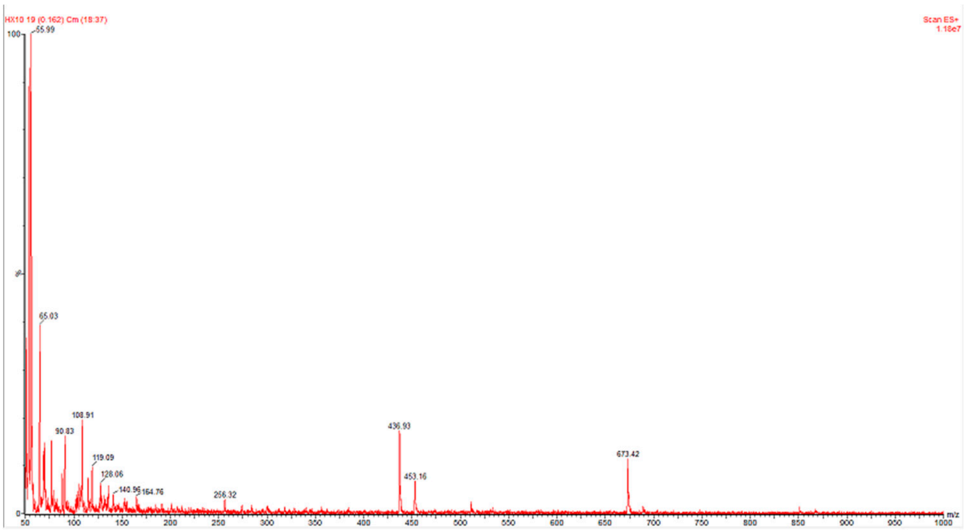

Figure S35. ES-MS of Compound **8**.

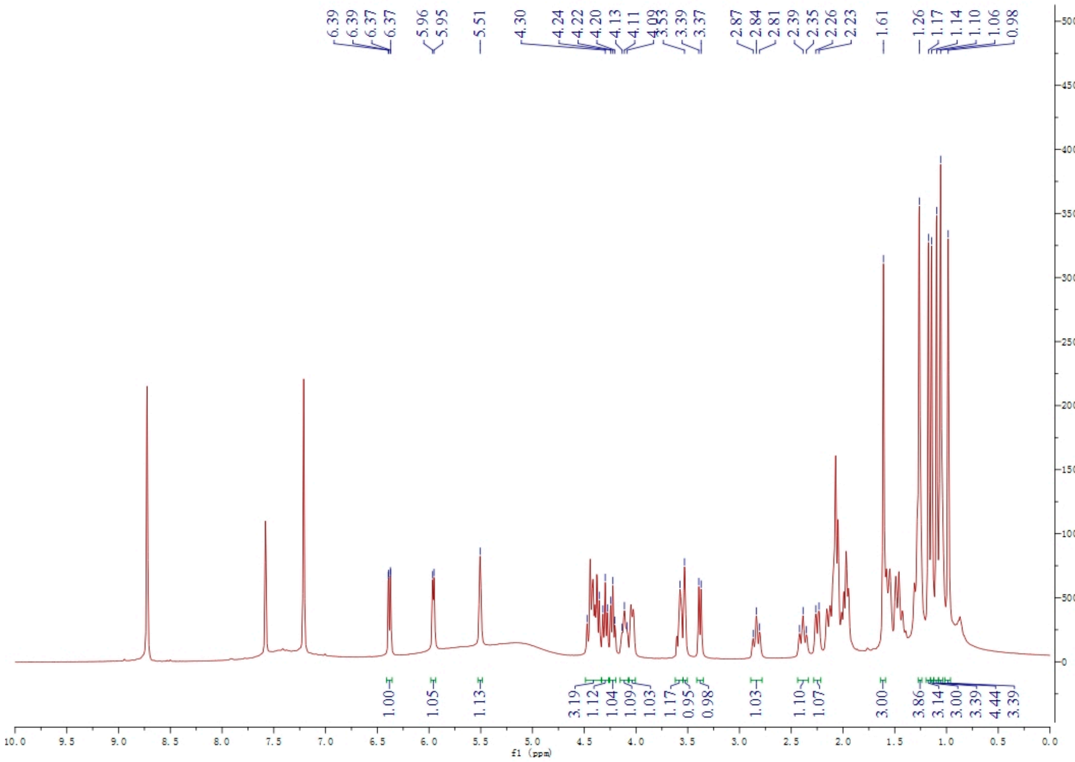

Figure S36. ^{1}H-NMR of Compound **8** (500 MHz, C5D5N).

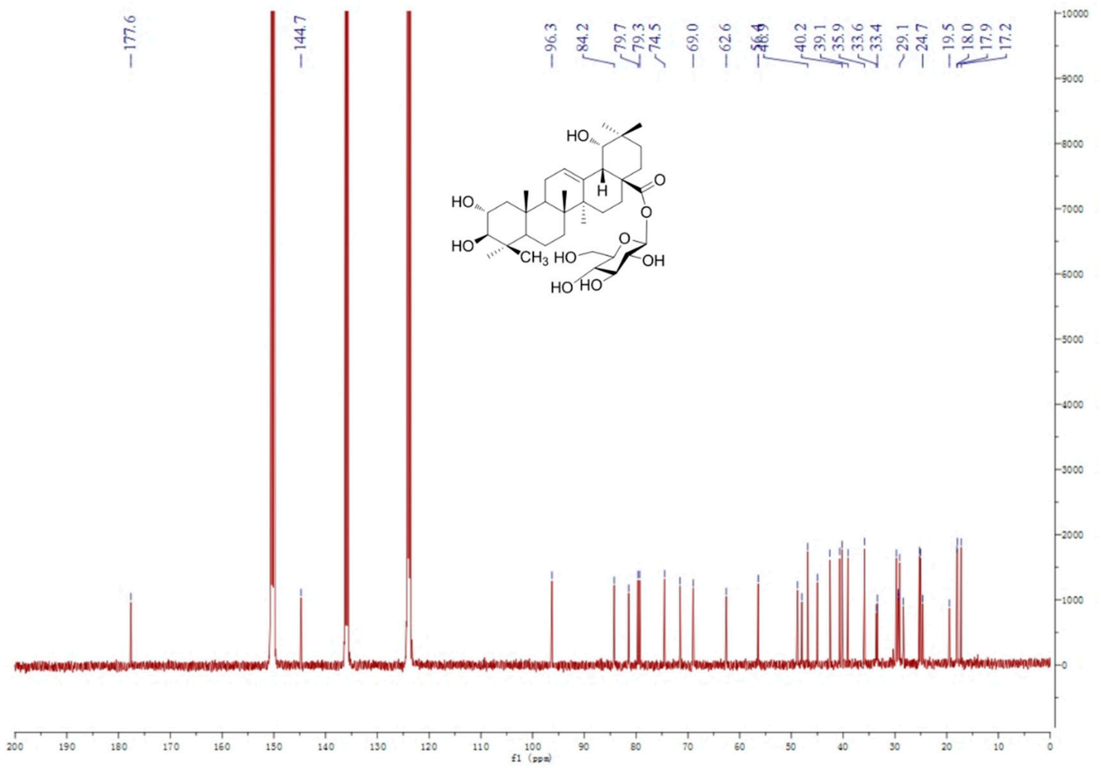

Figure S37. ^{13}C-NMR of Compound **8** (126 MHz, C5D5N).

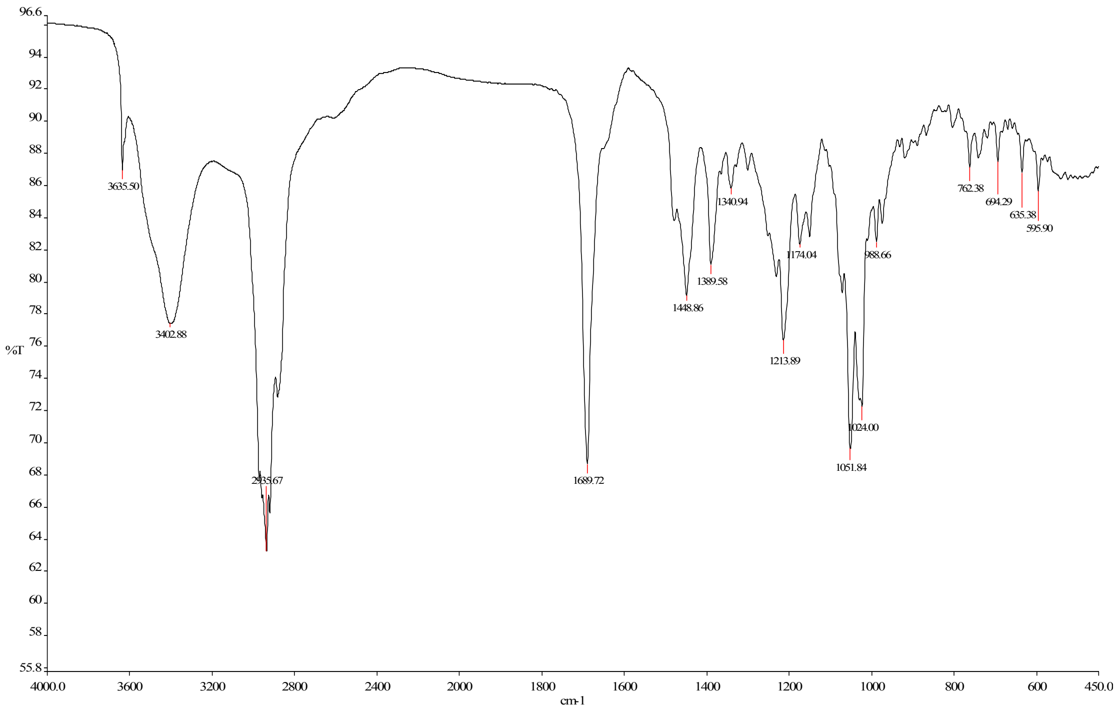

Figure S38. IR of Compound **9**.

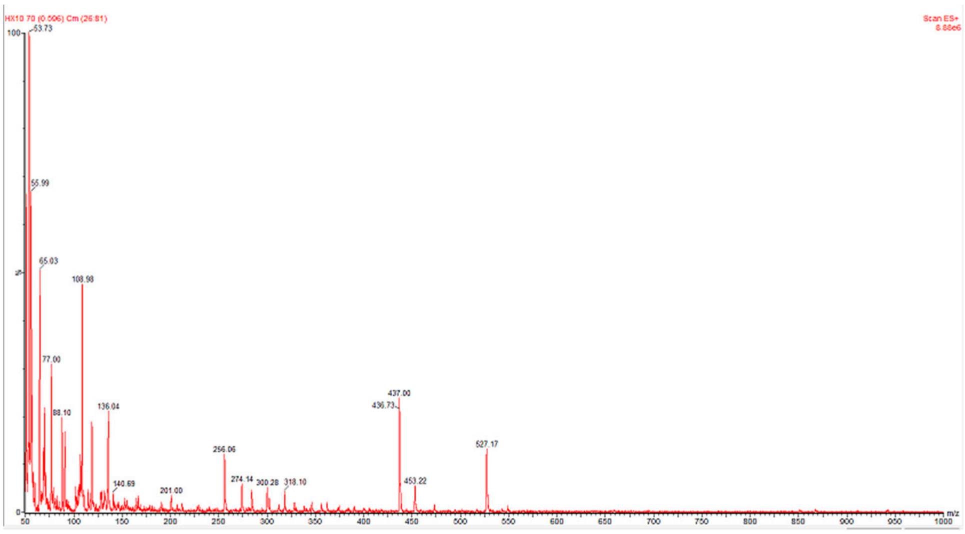

Figure S39. ES-MS of Compound **9**.

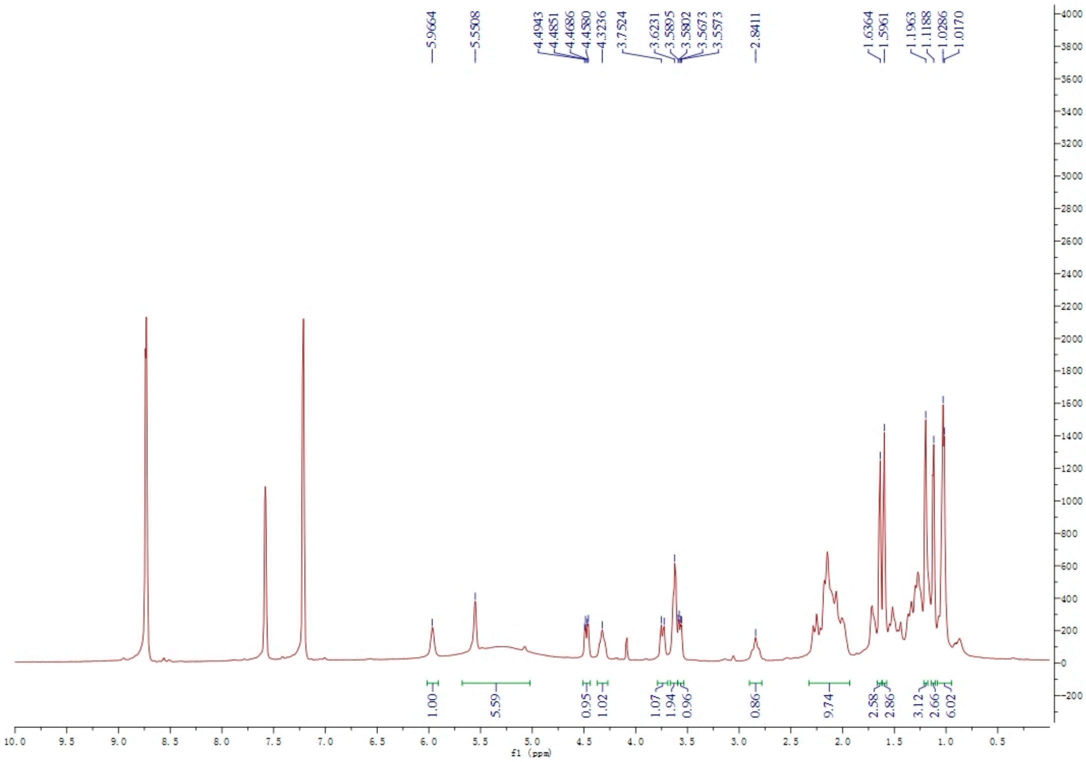

Figure S40. ^{1}H-NMR of Compound **9** (400 MHz, C5D5N).

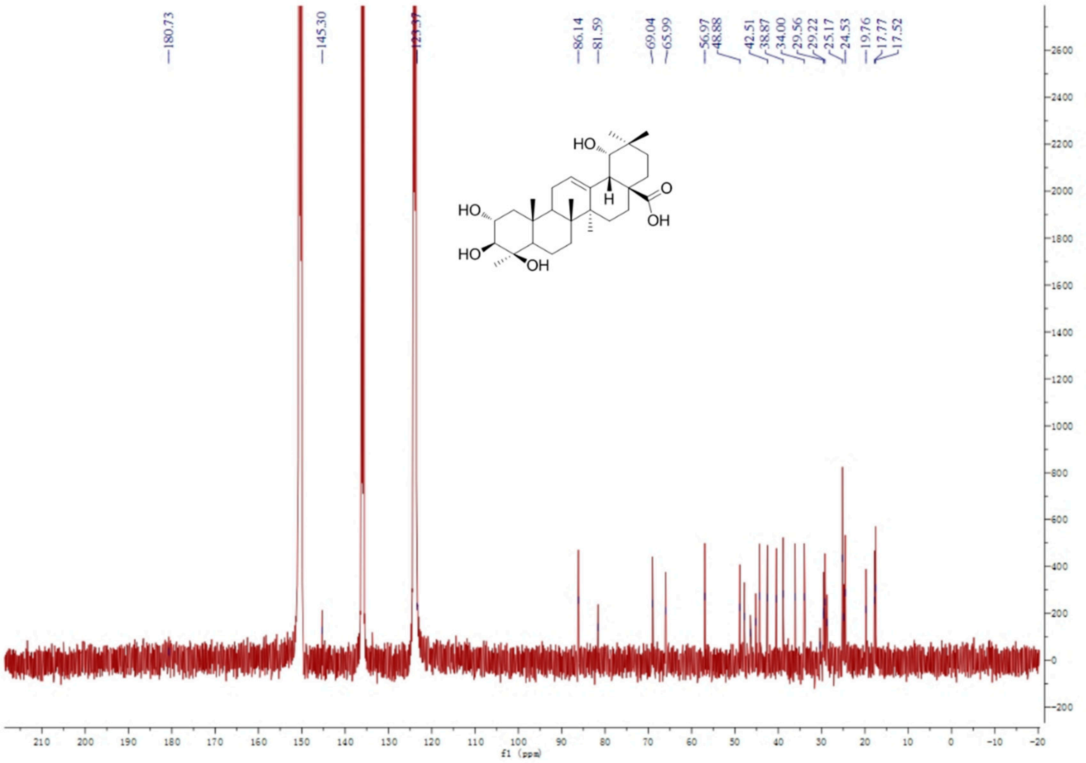

Figure S41. ^{13}C-NMR of Compound **9** (101 MHz, C5D5N).

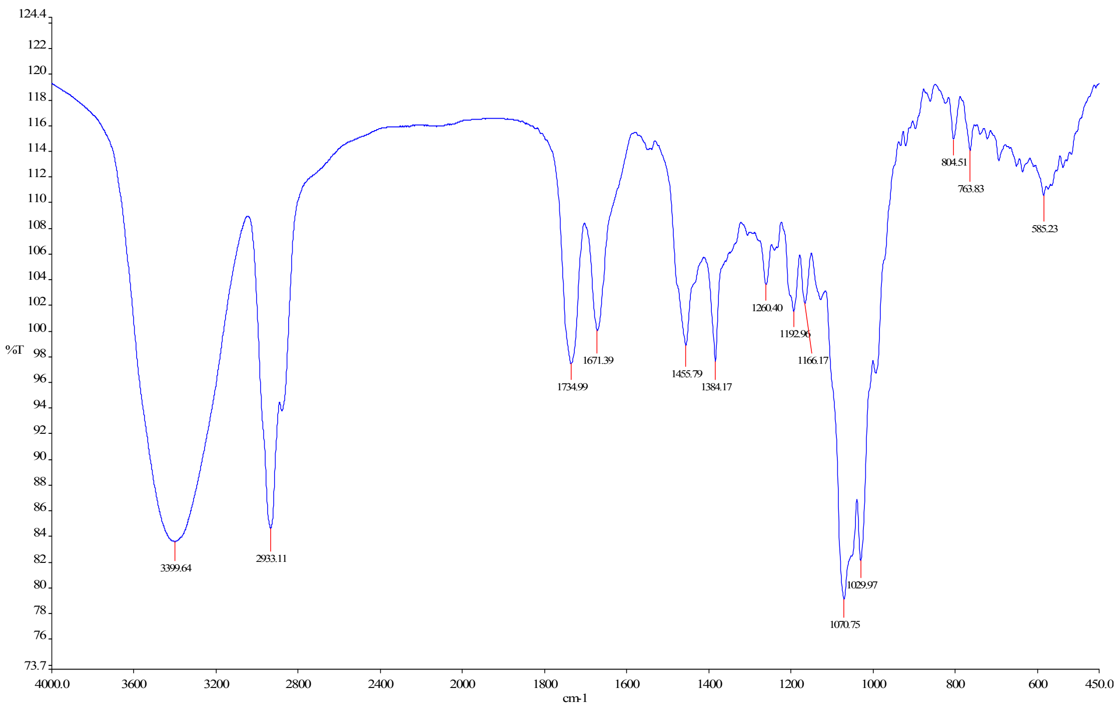

Figure S42. IR of Compound **10**.

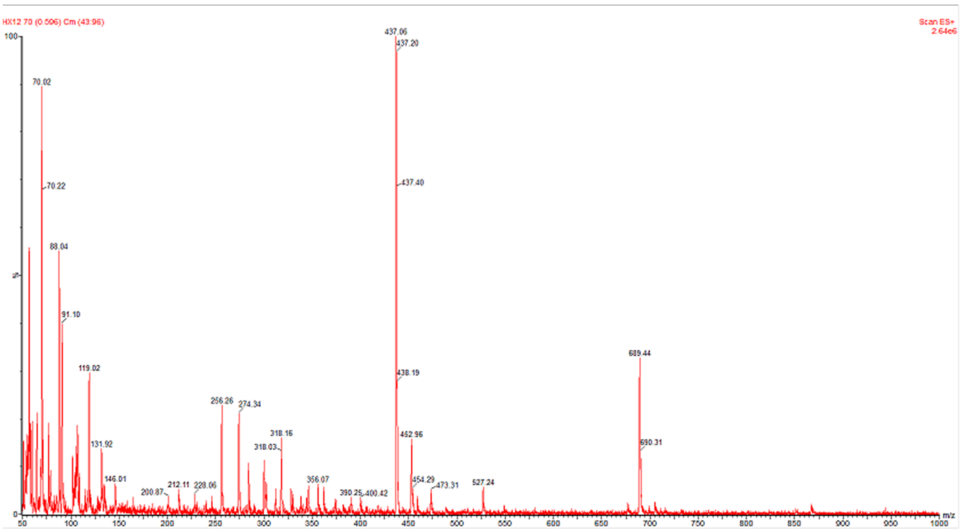

Figure S43. ES-MS of Compound **10**.

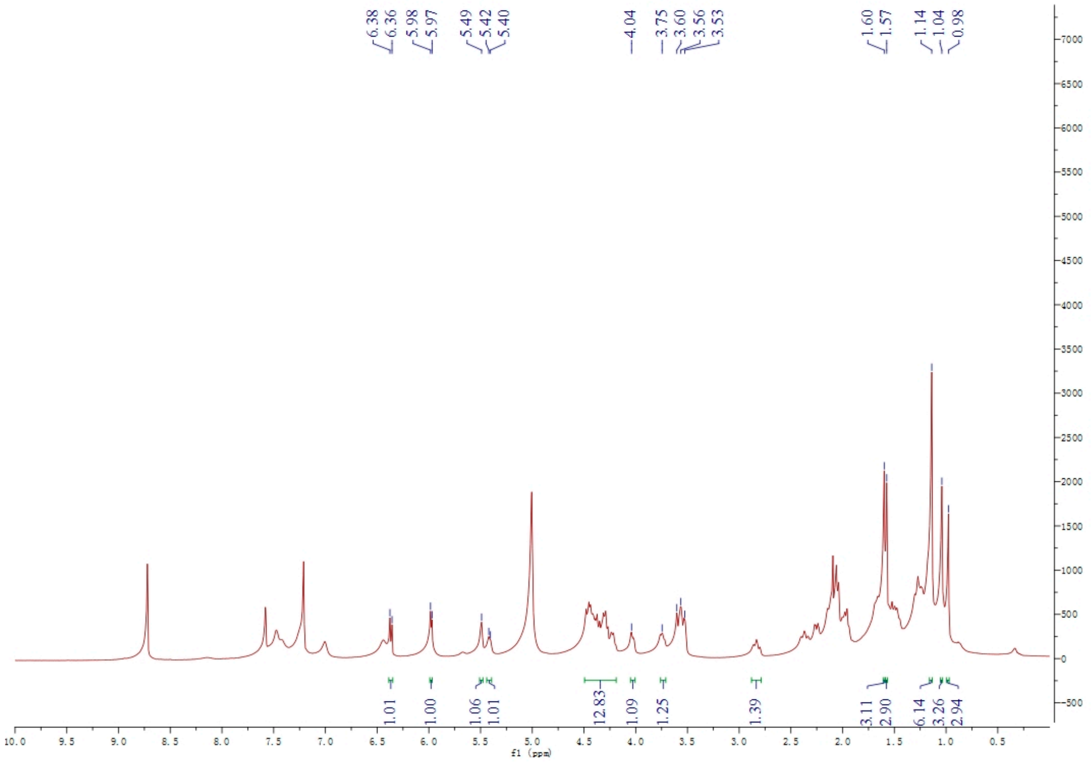

Figure S44. ^{1}H-NMR of Compound **10** (500 MHz, C5D5N).

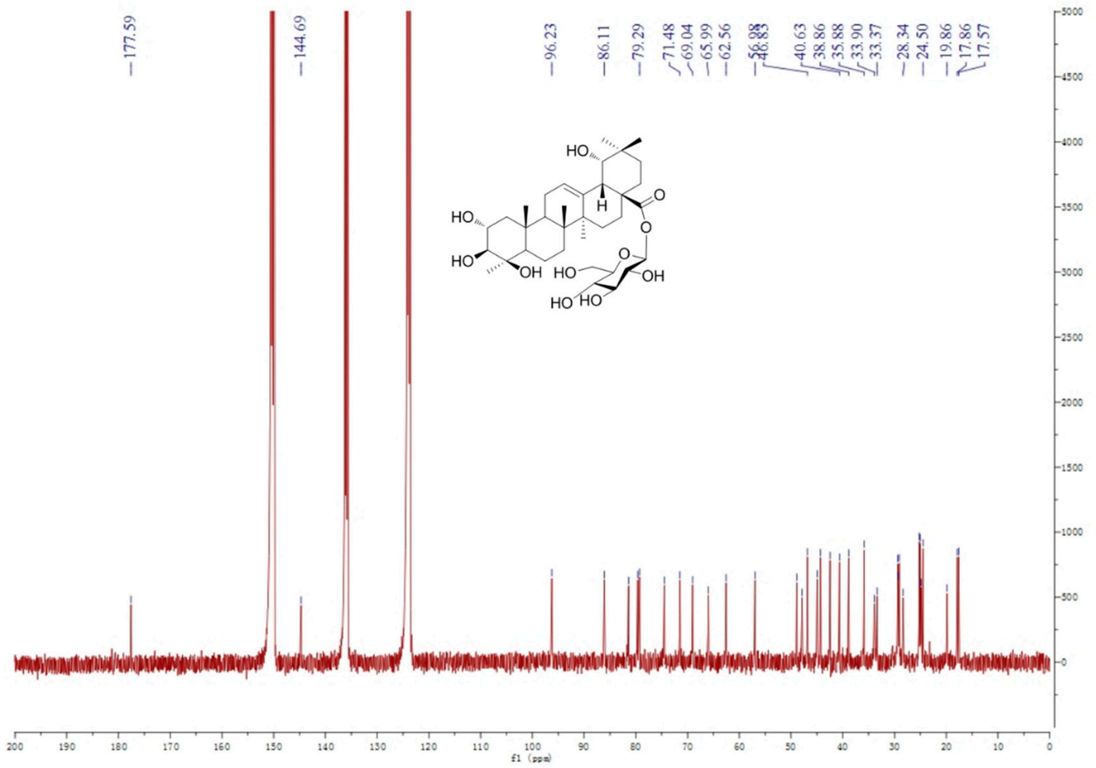

Figure S45. ^{13}C-NMR of Compound **10** (126 MHz, C5D5N).

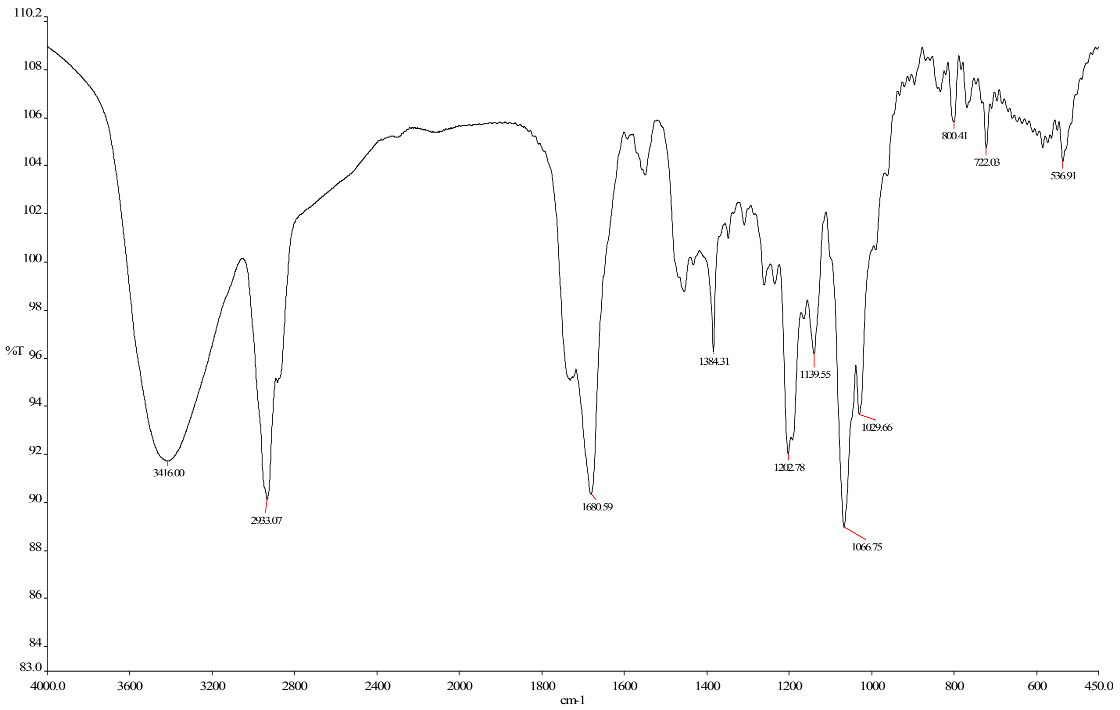

Figure S46. IR of Compound **11**.

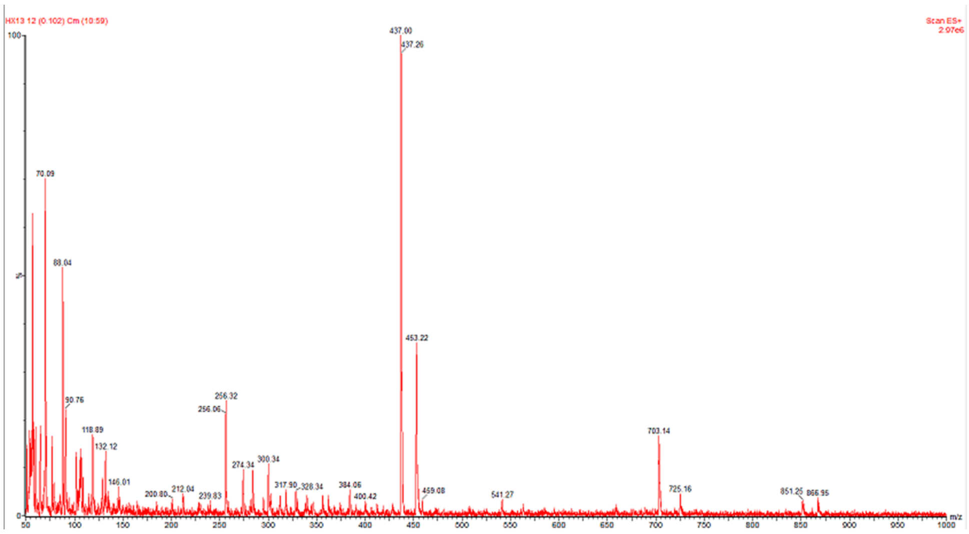

Figure S47. ES-MS of Compound **11**.

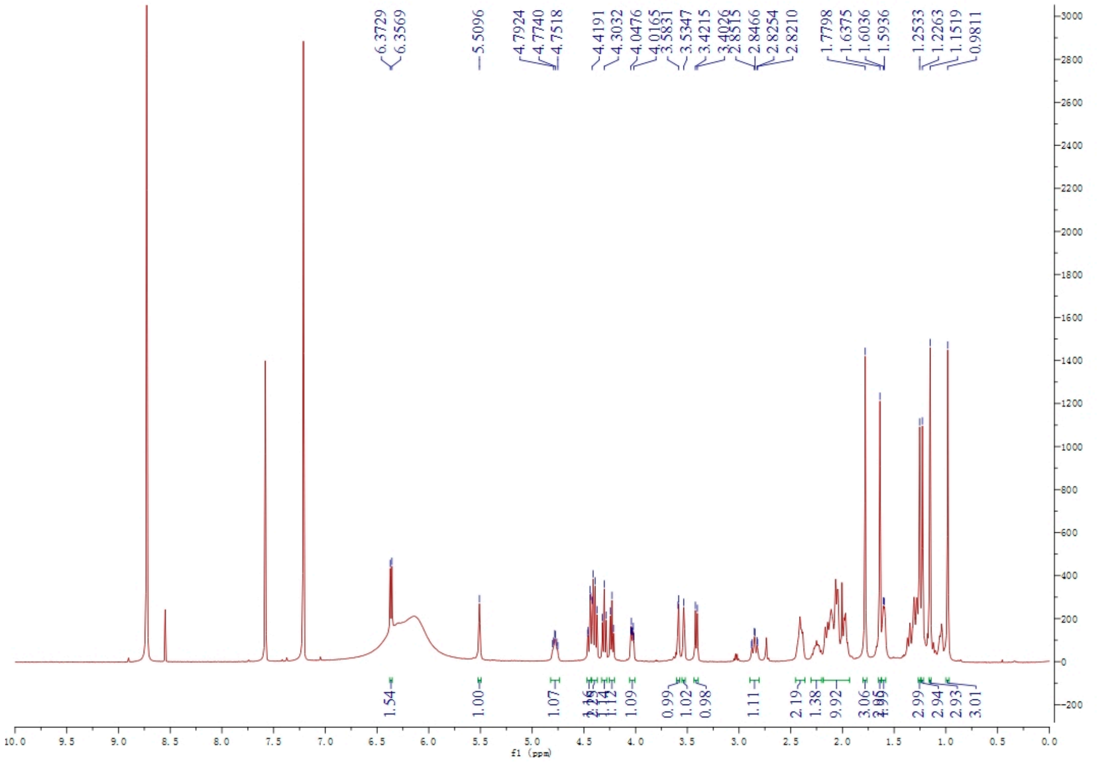

Figure S48. ^{1}H-NMR of Compound **11** (500 MHz, C5D5N).

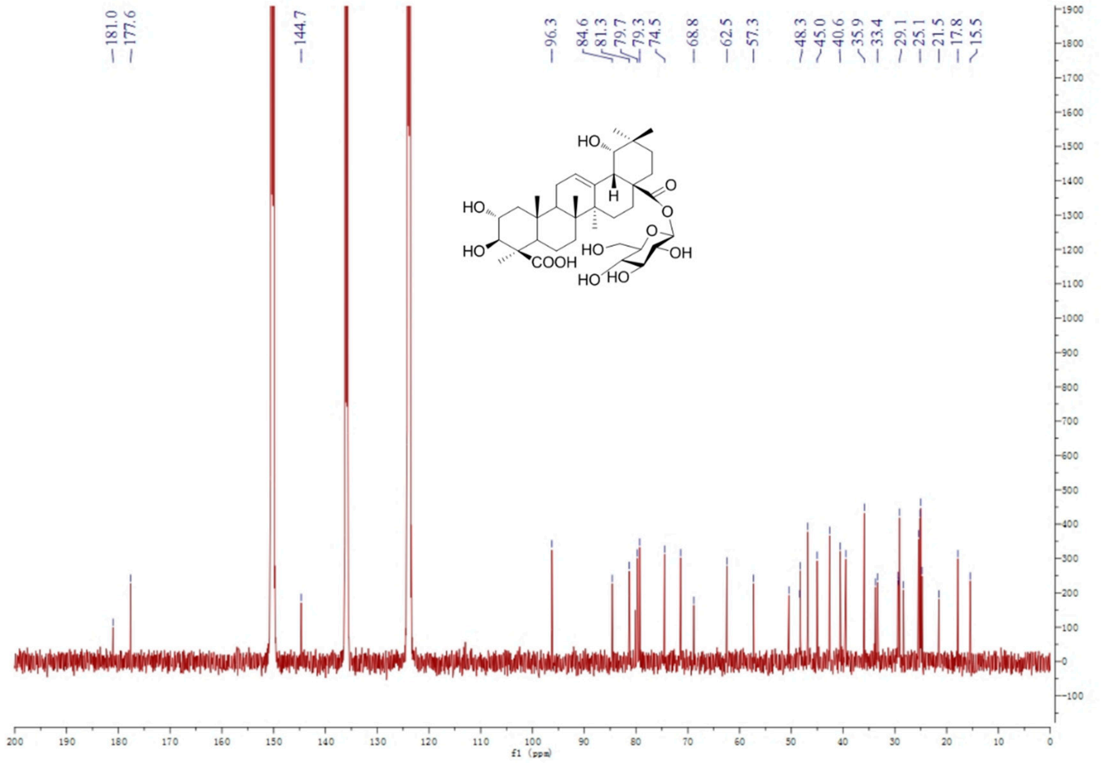

Figure S49. ^{13}C-NMR of Compound **11** (126 MHz, C5D5N).
